# A Systematic Review on Healthcare Analytics: Application and Theoretical Perspective of Data Mining

**DOI:** 10.3390/healthcare6020054

**Published:** 2018-05-23

**Authors:** Md Saiful Islam, Md Mahmudul Hasan, Xiaoyi Wang, Hayley D. Germack, Md Noor-E-Alam

**Affiliations:** 1 Mechanical and Industrial Engineering, Northeastern University, Boston, MA 02115, USA; islam.m@husky.neu.edu (M.S.I.); hasan.mdm@husky.neu.edu (M.M.H.); wang.xiaoyi@husky.neu.edu (X.W.); hayley.germack@yale.edu (H.D.G.); 2 National Clinician Scholars Program, Yale University School of Medicine, New Haven, CT 06511, USA; 3 Bouvé College of Health Sciences, Northeastern University, Boston, MA 02115, USA

**Keywords:** healthcare, data analytics, data mining, big data, healthcare informatics, literature review

## Abstract

The growing healthcare industry is generating a large volume of useful data on patient demographics, treatment plans, payment, and insurance coverage—attracting the attention of clinicians and scientists alike. In recent years, a number of peer-reviewed articles have addressed different dimensions of data mining application in healthcare. However, the lack of a comprehensive and systematic narrative motivated us to construct a literature review on this topic. In this paper, we present a review of the literature on healthcare analytics using data mining and big data. Following Preferred Reporting Items for Systematic Reviews and Meta-Analyses (PRISMA) guidelines, we conducted a database search between 2005 and 2016. Critical elements of the selected studies—healthcare sub-areas, data mining techniques, types of analytics, data, and data sources—were extracted to provide a systematic view of development in this field and possible future directions. We found that the existing literature mostly examines analytics in clinical and administrative decision-making. Use of human-generated data is predominant considering the wide adoption of Electronic Medical Record in clinical care. However, analytics based on website and social media data has been increasing in recent years. Lack of prescriptive analytics in practice and integration of domain expert knowledge in the decision-making process emphasizes the necessity of future research.

## 1. Introduction

Healthcare is a booming sector of the economy in many countries [1]. With its growth, come challenges including rising costs, inefficiencies, poor quality, and increasing complexity [2]. U.S. healthcare expenditures increased by 123% between 2010 and 2015—from $2.6 trillion to $3.2 trillion [3]. Inefficient—non-value added tasks (e.g., readmissions, inappropriate use of antibiotics, and fraud)—constitutes 21–47% of this enormous expenditure [4]. Some of these costs were associated with low quality care—researchers found that approximately 251,454 patients in the U.S. die each year due to medical errors [5]. Better decision-making based on available information could mitigate these challenges and facilitate the transition to a value-based healthcare industry [4]. Healthcare institutions are adopting information technology in their management system [6]. A large volume of data is collected through this system on a regular basis. Analytics provides tools and techniques to extract information from this complex and voluminous data [2] and translate it into information to assist decision-making in healthcare.

Analytics is the way of developing insights through the efficient use of data and application of quantitative and qualitative analysis [7]. It can generate fact-based decisions for “planning, management, measurement, and learning” purposes [2]. For instance, the Centers for Medicare and Medicaid Services (CMS) used analytics to reduce hospital readmission rates and avert $115 million in fraudulent payment [8]. Use of analytics—including data mining, text mining, and big data analytics—is assisting healthcare professionals in disease prediction, diagnosis, and treatment, resulting in an improvement in service quality and reduction in cost [9]. According to some estimates, application of data mining can save $450 billion each year from the U.S. healthcare system [10]. In the past ten years, researchers have studied data mining and big data analytics from both applied (e.g., applied to pharmacovigilance or mental health) and theoretical (e.g., reflecting on the methodological or philosophical challenges of data mining) perspectives.

In this review, we systematically organize and summarize the published peer-reviewed literature related to the applied and theoretical perspectives of data mining. We classify the literature by types of analytics (e.g., descriptive, predictive, prescriptive), healthcare application areas (i.e., clinical decision support, mental health), and data mining techniques (i.e., classification, sequential pattern mining); and we report the data source used in each review paper which, to our best knowledge, has never done before.

### Motivation and Scope

There is a large body of recently published review/conceptual studies on healthcare and data mining. We outline the characteristics of these studies—e.g., scope/healthcare sub-area, timeframe, and number of papers reviewed—in Table 1. For example, one study reviewed awareness effect in type 2 diabetes published between 2001 and 2005, identifying 18 papers [11]. This current review literature is limited—most of the papers listed in Table 1 did not report the timeframe and/or number of papers reviewed (expressed as N/A).

There is no comprehensive review available which presents the complete picture of data mining application in the healthcare industry. The existing reviews (16 out of 21) are either focused on a specific area of healthcare, such as clinical medicine (three reviews) [16,17,19], adverse drug reaction signal detection (two reviews) [25,26], big data analytics (four reviews) [8,10,22,24], or the application and performance of data mining algorithms (five reviews) [9,13,14,20,21]. Two studies focused on specific diseases (diabetes [11], skin diseases [18]). To the best of our knowledge, none of these studies present the universe of research that has been done in this field. These studies are also limited in the rigor of their methodology except for four articles [11,16,22,25], which provide key insights including the timeframe covered in the study, database search, and literature inclusion or exclusion criteria, but they are limited in their scope of topics covered (see Table 1).

Beyond condensing the applied literature, our review also adds to the body of theoretical reviews in the analytics literature. Current theoretical reviews are limited to methodological challenges and techniques to overcome those challenges [15,16,27] and application and impact of big data analytics in healthcare [23]. In summary, the current reviews listed in Table 1 lacks in (1) width of coverage in terms of application areas, (2) breadth of data mining techniques, (3) assessment of literature quality, and (4) systematic selection and analysis of papers. In this review, we aim to fill the above-mentioned gaps. We add to this literature by covering the applied and theoretical perspective of data mining and big data analytics in healthcare with a more comprehensive and systematic approach.

## 2. Methodology

The methodology of our review followed the checklist proposed by the Preferred Reporting Items for Systematic Reviews and Meta-Analyses (PRISMA) [28]. We assessed the quality of the selected articles using JBI Critical Appraisal Checklist for analytical cross sectional studies [29] and Critical Appraisal Skills Programme (CASP) qualitative research checklist [30].

### 2.1. Input Literature

**Figure 1:**
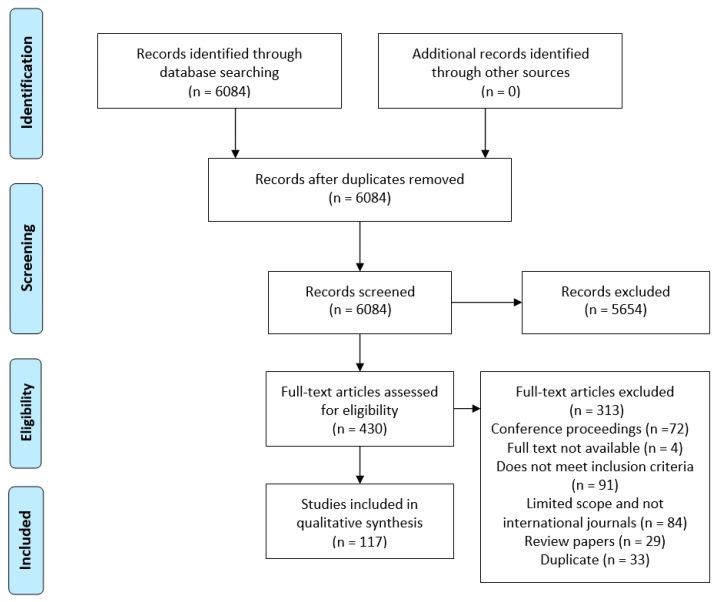
Preferred Reporting Items for Systematic Reviews and Meta-Analyses (PRISMA) flow chart [28] illustrating the literature search process.

Selected literature and their selection process for the review are described in this section. Initially a two phase advance keyword search was conducted on the database Web of Science and one phase (Phase 2) search in PubMed and Google Scholar with time filter 1 January 2005 to 31 December 2016 in “All Fields”. Journal articles written in English was added as additional filters. Keywords listed in Table 2 were used in different phases. The complete search procedure was conducted using the following procedure:

- Inclusion criteria: The phase 1 search resulted in thousands of articles which was then narrowed down using the phase 2 keywords within the initial search space. Second phase resulted in 129 articles in Web of Science, and 5255 articles in PubMed. Search in Google Scholar search engine was conducted with phase 2 keywords which resulted in 700 articles. The title, abstract, and keywords of those articles were screened and those *discussing the application of data mining and big data in the healthcare decision-making process* were retained for full-text review. To make the screening process efficient, duplicate articles were removed at the eligibility phase instead of screening phase of the PRISMA review process (Figure 1).
- Exclusion criteria: This included articles reporting on results of: *qualitative study, survey, focus group study, feasibility study, monitoring device, team relationship measurement, job satisfaction, work environment, “what-if” analysis, data collection technique, editorials or short report, merely mention data mining, and articles not published in international journals*. Duplicates were removed (33 articles). Finally, 117 articles were retained for the review. Figure 1 provides a PRISMA [28] flow diagram of the review process and Supplementary Information File S1 (Table S1) provides the PRISMA checklist.

### 2.2. Quality Assessment and Processing Steps

The full text of each of the 117 articles was reviewed separately by two researchers to eliminate bias [28]. To assess the quality of the cross sectional studies, we applied the JBI Critical Appraisal Checklist for Analytical Cross Sectional Studies [29]. For theoretical papers, we applied the Critical Appraisal Skills Programme (CASP) qualitative research checklist [30]. We modified the checklist items, as not all items specified in the JBI or CASP checklists were applicable to studies on healthcare analytics (Supplementary Materials Table S2). We evaluated each article’s quality based on inclusion of: (1) clear objective and inclusion criteria; (2) detailed description of sample population and variables; (3) data source (e.g., hospital, database, survey) and format (e.g., structured Electronic Medical Record (EMR), International Classification of Diseases code, unstructured text, survey response); (4) valid and reliable data collection; (5) consideration of ethical issues; (6) detailed discussion of findings and implications; (7) valid and reliable measurement of outcomes; and (8) use of an appropriate data mining tool for cross-sectional studies and (1) clear statement of aims; (2) appropriateness of qualitative methodology; (3) appropriateness of research design; (4) clearly stated findings; and (5) value of research for the theoretical papers. Summary characteristics from any study fulfilling these criteria were included in the final data aggregation (Supplementary Materials Table S3).

**Figure 2:**
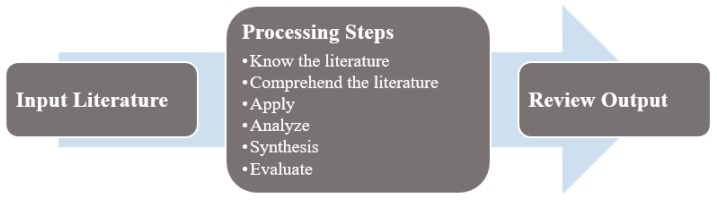
Three stages of effective literature review process, adapted from Levy and Ellis [31].

**Figure 3:**
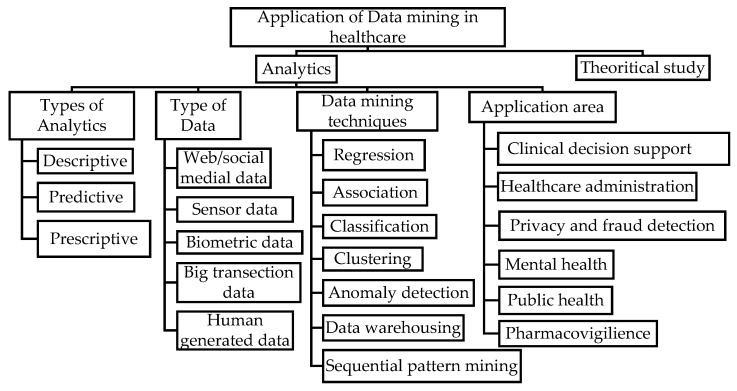
Classification scheme of the literature.

To summarize the body of knowledge, we adopted the three-step processing methodology outlined by Levy and Ellis [31] and Webster and Watson [32] (Figure 2). During the review process, information was extracted by identifying and defining the problem, understanding the solution process and listing the important findings (“Know the literature”). We summarized and compared each article with the articles associated with the similar problems (“Comprehend the literature”). This simultaneously ensured that any irrelevant information was not considered for the analysis. The summarized information was stored in a spreadsheet in the form of a concept matrix as described by Webster and Watson [32]. We updated the concept matrix periodically, after completing every 20% of the articles which is approximately 23 articles, to include new findings (“Apply”). Based on the concept matrix, we developed a classification scheme (see Figure 3) for further comparison and contrast. We established an operational definition (see Table 3) for each class and same class articles were separated from the pool (“Analyze and Synthesis”). We compared classifications between researchers and we resolved disagreements (on six articles) by discussion. The final classification provided distinguished groups of articles with summary, facts, and remarks made by the reviewers (“Evaluate”).

### 2.3. Results

**Figure 4:**
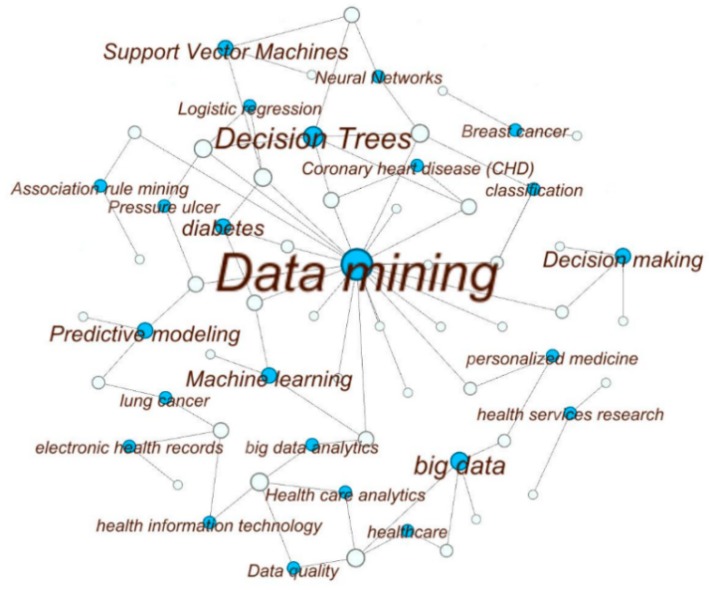
Visualization of high-frequency keywords of the reviewed papers. The white circles symbolize the articles and the blue circles represent keywords. The keywords that occurred only once are eliminated as well as the corresponding articles. The size of the blue circles and the texts represent how often that keyword is found. The size of the white circles is proportional to the number of keywords used in that article. The links represents the connections between the keywords and the articles. For example, if a blue circle has three links (e.g., Decision-Making) that means that keyword was used in three articles. The diagram is created with the open source software Gephi [34].

The network diagram of selected articles and the keywords listed by authors in Figure 4 represents the outcome of the methodological review process. We elaborate on the resulting output in the subsequent sections using the structure of the developed classification scheme (Figure 3). We also report the potential future research areas.

#### 2.3.1. Methodological Quality of the Studies

Out of 117 papers included in this review, 92 applied analytics and 25 were qualitative/conceptual. The methodological quality of the analytical studies (92 out of 117) were evaluated by a modified version of 8 yes/no questions suggested in JBI Critical Appraisal Checklist for Analytical Cross Sectional Studies [29]. Each question contains 1 point (1 if the answer is Yes or 0 for No). The score achieved by each paper is provided in the final column of Supplementary Materials Table S3. On average, each paper applying analytics scored 7.6 out of 8, with a range of 6–8 points. Major drawbacks were the absence of data source and performance measure of data mining algorithms. Out of 92 papers, 23 did not evaluate or mention the performance of the applied algorithms and eight did not mention the source of the data. However, all the papers in healthcare analytics had a clear objective and a detailed discussion of sample population and variables. Data used in each paper was either de-identified/anonymized or approved by institute’s ethical committee to ensure patient confidentiality.

We applied the Critical Appraisal Skills Programme (CASP) qualitative research checklist [30] to evaluate the quality of the 25 theoretical papers. Five questions (out of ten) in that checklist were not applicable to the theoretical studies. Therefore, we evaluated the papers in this section in a five-point scale (1 if the answer is Yes or 0 for No). Papers included in this review showed high methodological quality as 21 papers (out of 25) scored 5. The last column in the Supplementary Materials Table S3 provides the score achieved by individual papers.

#### 2.3.2. Distribution by Publication Year

**Figure 5:**
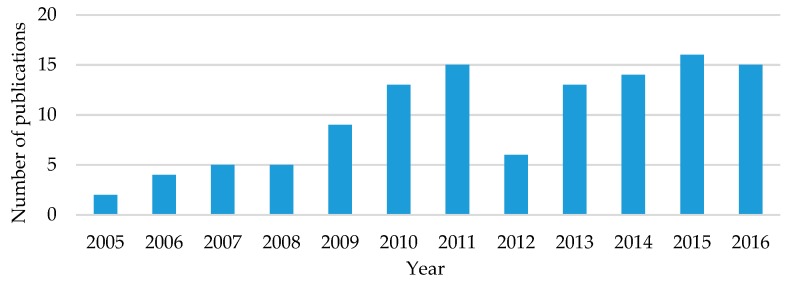
Distribution of publication by year (117 articles).

The distribution of articles published related to data mining and big data analytics in healthcare across the timeline of the study (2005–2016) is presented in Figure 5. The distribution shows an upward trend with at least two articles in each year and more than ten articles in the last four years. Additionally, this trend represents the growing interest of government agencies, healthcare practitioners, and academicians in this interdisciplinary field of research. We anticipate that the use of analytics will continue in the coming years to address rising healthcare costs and need of improved quality of care.

#### 2.3.3. Distribution by Journal

Articles published in 74 different journals were included in this study. Table 4 lists the top ten journals in terms of number of papers published. *Expert System with Application* was the dominant source of literature on data mining application in healthcare with 7 of the 117 articles. Journals were interdisciplinary in nature and spanned computational journals like *IEEE Transection on Information Technology in Biomedicine* to policy focused journal like *Health Affairs*. Articles published in *Expert System with Application, Journal of Medical Systems, Journal of the American Medical Informatics Association, Healthcare Informatics Research* were mostly related to analytics applied in clinical decision-making and healthcare administration. On the other hand, articles published in *Health Affairs* were predominantly conceptual in nature addressing policy issues, challenges, and potential of this field.

## 3. Healthcare Analytics

Out of 117 articles, 92 applied analytics for decision-making in healthcare. We discuss the types of analytics, the application area, the data, and the data mining techniques used in these articles and summarize them in Supplementary Materials Table S4.

### 3.1. Types of Analytics

**Figure 6:**
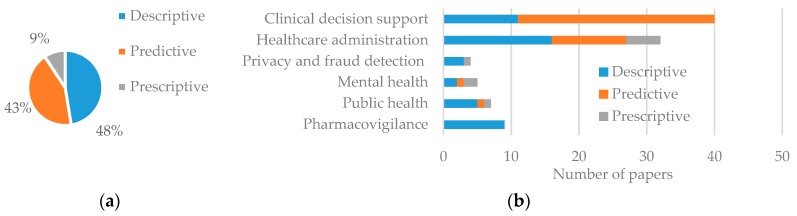
Types of analytics used in literature. (**a**) Percentage of analytics type; (**b**) Analytics type by application area.

We identified three types of analytics in the literature: descriptive (i.e., exploration and discovery of information in the dataset), predictive (i.e., prediction of upcoming events based on historical data) and prescriptive (i.e., utilization of scenarios to provide decision support). Five of the 92 studies employed both descriptive and predictive analytics. In Figure 6, which displays the percentage of healthcare articles using each analytics type, we show that descriptive analytics is the most commonly used in healthcare (48%). Descriptive analytics was dominant in all the application areas except in clinical decision support. Among the application areas, pharmacovigilance studies only used descriptive analytics as this application area is focused on identifying an association between adverse drug effects with medication. Predictive analytics was used in 43% articles. Among application areas, clinical decision support had the highest application of predictive analytics as many studies in this area are involved in risk and morbidity prediction of chest pain, heart attack, and other diseases. In contrast, use of prescriptive analytics was very uncommon (only 9%) as most of these studies were focused on either a specific population base or a specific disease scenario. However, some evidence of prescriptive analytics was found in public healthcare, administration, and mental health (see Supplementary Materials Table S4). These studies create a data repository and/or analytical platform to facilitate decision-making for different scenarios.

### 3.2. Types of Data

**Figure 7:**
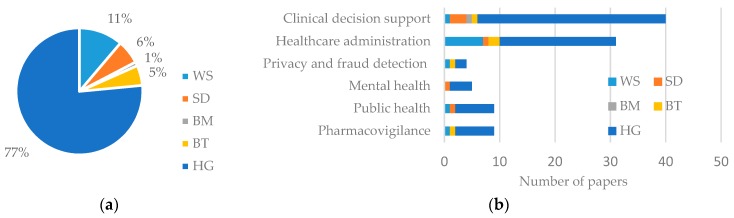
Percentage of data type used (**a**) and type of data used by application area (**b**).

To identify types of data, we adopted the classification scheme identified by Raghupathi and Raghupathi [23] which takes into account the nature (i.e., text, image, number, electronic signal), source, and collection method of data together. Table 3 provides the operational definitions of taxonomy adopted in this paper. Figure 7a presents the percentage of data type used and Figure 7b, the number of usage by application area. As expected, human generated (HG) data, including EMR, Electronic Health Record (HER), and Electronic Patient Record (EPR), is the most commonly (77%) used form. Web or Social media (WS) data is the second dominant (11%) type of data, as increasingly more people are using social media now and ongoing digital revolution in the healthcare sector [35]. In addition, recent development in Natural Language Processing (NLP) techniques is making the use of WS data easier than before [36]. The other three types of data (SD, BT, and BM) consist of only about 12% of total data usage, but popularity and market growth of wearable personal health tracking devices [37] may increase the use of SD and BM data.

### 3.3. Data Mining Techniques

**Figure 8:**
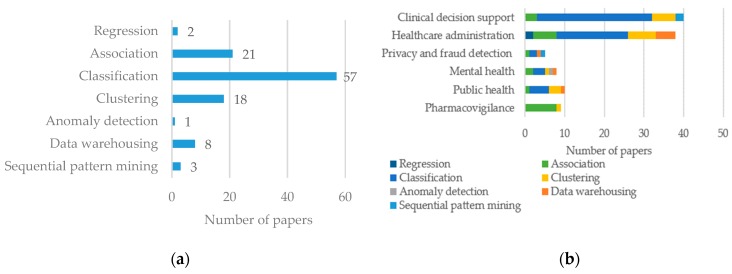
Utilization of data mining techniques, (**a**) by percentage and (**b**) by application area.

Data mining techniques used in the articles reviewed include classification, clustering, association, anomaly detection, sequential pattern mining, regression, and data warehousing. While elaborate description of each technique and available algorithms is out of scope of this review, we report the frequency of each technique and its sector wise distribution in Figure 8a,b, respectively. Among the articles included in the review, 57 used classification techniques to analyze data. Association and clustering were used in 21 and 18 articles, respectively. Use of other techniques was less frequent.

A high proportion (8 out of 9) of pharmacovigilance papers used association. Use of classification was dominant in every sector except pharmacovigilance (Figure 8b). Data warehousing was mostly used in healthcare administration (Figure 8b).

**Figure 9:**
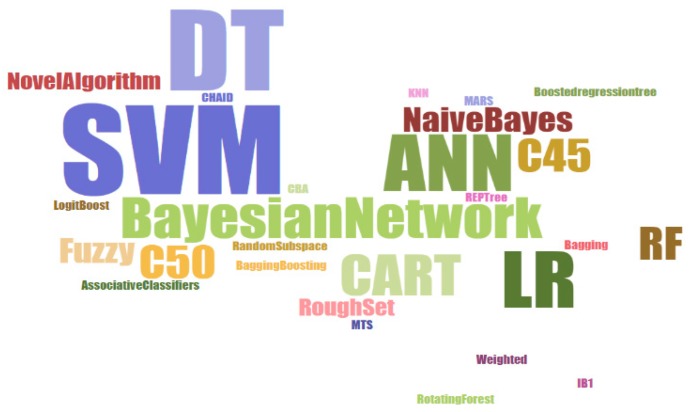
Word cloud [39] with classification algorithms.

We delved deeper into classification as it was utilized in the majority (57 out of 92) of the papers. There are a number of algorithms used for classification, which we present in a word cloud in Figure 9. Support Vector Machine (SVM), Artificial Neural Network (ANN), Logistic Regression (LR), Decision Tree (DT), and DT based algorithms were the most commonly used. Random Forest (RF), Bayesian Network and Fuzzy-based algorithms were also often used. Some papers (three papers) introduced novel algorithms for specific applications. For example, Yeh et al. [38] developed discrete particle swarm optimization based classification algorithm to classify breast cancer patients from a pool of general population. Self-organizing maps and K-means were the most commonly used clustering algorithm in healthcare. Performance (e.g., accuracy, sensitivity, specificity, area under the ROC curve, positive predictive value, negative predictive value etc.) of each of these algorithms varied by application and data type. We recommend applying multiple algorithms and choosing the one which achieves the best accuracy.

## 4. Application of Analytics in Healthcare

**Figure 10:**
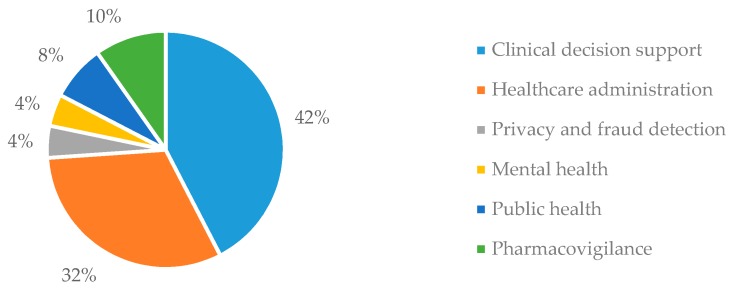
Percentage of papers utilized healthcare analytics by application area (92 articles out of 117).

Table 3 provides the operational definitions of the six application areas (i.e., clinical decision support, healthcare administration, privacy and fraud detection, mental health, public health, and pharmacovigilance) identified in this review. Figure 10 shows the percentage of articles in each area. Among different classes in healthcare analytics, data mining application is mostly applied in clinical decision support (42%) and administrative purposes (32%). This section discusses the application of data mining in these areas and identifies the main aims of these studies, performance gaps, and key features.

### 4.1. Clinical Decision Support

Clinical decision support consists of descriptive and/or predictive analysis mostly related to cardiovascular disease (CVD), cancer, diabetes, and emergency/critical care unit patients. Some studies developed novel data mining algorithms which we review. Table 5 describes the topics investigated and data sources used by papers using clinical decision-making, organized by major diseases category.

#### 4.1.1. Cardiovascular Disease (CVD)

CVD is one of the most common causes of death globally [45,77]. Its public health relevance is reflected in the literature—it was addressed by seven articles (18% of articles in clinical decision support).

Risk factors related to Coronary Heart Disease (CHD) were distilled into a decision tree based classification system by researchers [40]. The authors investigated three events: Coronary Artery Bypass Graft Surgery (CABG), Percutaneous Coronary Intervention (PCI), and Myocardial Infarction (MI). They developed three models: CABG vs. non-CABG, PCI vs. non-PCI, and MI VS non-MI. The risk factors for each event were divided into four groups in two stages. The risk factors were separated into before and after the event at the 1st stage and modifiable (e.g., smoking habit or blood pressure) and non-modifiable (e.g., age or sex) at the 2nd stage for each group. After classification, the most important risk factors were identified by extracting the classification rules. The Framingham equation [78]—which is widely used to calculate global risk for CHD was used to calculate the risk for each event. The most important risk factors identified were age, smoking habit, history of hypertension, family history, and history of diabetes. Other studies on CHD show similar results [79,80,81]. This study had implications for healthcare providers and patients by identifying risk factors to specifically target, identify and in the case of modifiable factors, reduce CHD risk [40].

Data mining has also been applied to diagnose Coronary Artery Disease (CAD) [41]. Researchers showed that in lieu of existing diagnostic methods (i.e., Coronary Angiography (CA))—which are costly and require high technical skill—data mining using existing data like demographics, medical history, simple physical examination, blood tests, and noninvasive simple investigations (e.g., heart rate, glucose level, body mass index, creatinine level, cholesterol level, arterial stiffness) is simple, less costly, and can be used to achieve a similar level of accuracy. Researchers used a four-step classification process: (1) Decision tree was used to classify the data; (2) Crisp classification rules were generated; (3) A fuzzy model was created by fuzzifying the crisp classifier rules; and (4) Fuzzy model parameters were optimized and the final classification was made. The proposed optimized fuzzy model achieved 73% of prediction accuracy and improved upon an existing Artificial Neural Network (ANN) by providing better interpretability.

Traditional data mining and machine learning algorithms (e.g., probabilistic neural networks and SVM) may not be advanced enough to handle the data used for CVD diagnosis, which is often uncertain and highly dimensional in nature. To tackle this issue, researchers [42] proposed a Fuzzy standard additive model (SAM) for classification. They used adaptive vector quantization clustering to generate unsupervised fuzzy rules which were later optimized (minimized the number of rules) by Genetic Algorithm (GA). They then used the incremental form of a supervised technique, Gradient Descent, to fine tune the rules. Considering the highly time consuming process of the fuzzy system given large number of features in the data, the number of features was reduced with wavelet transformation. The proposed algorithm achieved better accuracy (78.78%) than the probabilistic neural network (73.80%), SVM (74.27%), fuzzy ARTMAP (63.46%), and adaptive neuro-fuzzy inference system (74.90%). Another common issue in cardiovascular event risk prediction is the censorship of data (i.e., the patient’s condition is not followed up after they leave hospital and until a new event occurs; the available data becomes right-censored). Elimination and exclusion of the censored data create bias in prediction results. To address the censorship of the data in their study on CVD event risk prediction after time, two studies [43,44] used Inverse Probability Censoring Weighting (IPCW). IPCW is a pre-processing step used to calculate the weights on data which are later classified using Bayesian Network. One of these studies [43] provided an IPCW based system which is compatible with any machine learning algorithm.

Electrocardiography (ECG)—non-invasive measurement of the electrical activity of the heartbeat—is the most commonly used medical studies in the assessment of CVD. Machine learning offers potential optimization of traditional ECG assessment which requires decompressing before making any diagnosis. This process takes time and large space in computers. In one study, researchers [45] developed a framework for real-time diagnosis of cardiovascular abnormalities based on compressed ECG. To reduce diagnosis time—which is critical for clinical decision-making regarding appropriate and timely treatment—they proposed and tested a mobile based framework and applied it to wireless monitoring of the patient. The ECG was sent to the hospital server where the ECG signals were divided into normal and abnormal clusters. The system detected cardiac abnormality with 97% accuracy. The cluster information was sent to patient’s mobile phone; and if any life-threatening abnormality was detected, the mobile phone alerted the hospital or the emergency personnel.

Data analytics have also been applied to more rare CVDs. One study [46] developed an intervention prediction model for Hypoplastic Left Heart Syndrome (HLHS). HLHS is a rare form of fatal heart disease in infants, which requires surgery. Post-surgical evaluation is critical as patient condition can shift very quickly. Indicators of wellness of the patients are not easily or directly measurable, but inferences can be made based on measurable physiological parameters including pulse, heart rhythm, systemic blood pressure, common atrial filling pressure, urine output, physical exam, and systemic and mixed venous oxygen saturations. A subtle physiological shift can cause death if not noticed and intervened upon. To help healthcare providers in decision-making, the researchers developed a prediction model by identifying the correlation between physiological parameters and interventions. They collected 19,134 records of 17 patients in Pediatric Intensive Care Units (PICU). Each record contained different physiological parameters measured by devices and noted by nurses. For each record, a wellness score was calculated by the domain experts. After classifying the data using a rough set algorithm, decision rules were extracted for each wellness score to aid in making intervention plans. A new measure for feature selection—Combined Classification Quality (CCQ)—was developed by considering the effect of variations in a feature values and distinct outcome each feature value leads to. Authors showed that higher value of CCQ leads to higher classification accuracy which is not always true for commonly used measure classification quality (CQ). For example, two features with CQ value of 1 leads to very different classification accuracy—35.5% and 75%. Same two features had CCQ value 0.25 and 0.40, features with 0.40 CCQ produced 75% classification accuracy. By using CCQ instead of CQ, researchers can avoid such inconsistency.

#### 4.1.2. Diabetes

The disease burden related to diabetes is high and rising in every country. According to the World Health Organization’s (WHO) prediction, it will become the seventh leading cause of death by 2030 [82]. Data mining has been applied to identify rare forms of diabetes, identify the important factors to control diabetes, and explore patient history to extract knowledge. We reviewed 7 studies that applied healthcare analytics to diabetes.

Researchers extracted knowledge about diabetes treatment pathways and identified rare forms and complications of diabetes using a three level clustering framework from examination history of diabetic patients [48]. In this three-level clustering framework, the first level clustered patients who went through regular tests for monitoring purposes (e.g., checkup visit, glucose level, urine test) or to diagnose diabetes-related complications (e.g., eye tests for diabetic retinopathy). The second level explored patients who went through diagnosis for specific or different diabetic complications only (e.g., cardiovascular, eye, liver, and kidney related complications). These two level produced 2939 outliers out of 6380 patients. At the third level, authors clustered these outlier patients to gain insight about rare form of diabetes or rare complications. A density based clustering algorithm, DBSCAN, was used for clustering as it doesn’t require to specify the number of clusters apriori and is less sensitive to noise and outliers. This framework for grouping patients by treatment pathway can be utilized to evaluate treatment plans and costs. Another group of researchers [49] investigated the important factors related to type 2 diabetes control. They used feature selection via supervised model construction (FSSMC) to select the important factors with rank/order. They applied naïve bayes, IB1 and C4.5 algorithm with FSSMC technique to classify patients having poor or good diabetes control and evaluate the classification efficiency for different subsets of features. Experiments performed with physiological and laboratory information collected from 3857 patients showed that the classifier algorithms performed best (1–3% increase in accuracy) with the features selected by FSSMC. Age, diagnosis duration, and Insulin treatment were the top three important factors.

Data analytics have also been applied to identify patients with type 2 diabetes. In one study [52], using fragmented data from two different healthcare centers, researchers evaluated the effect of data fragmentation on a high throughput clinical phenotyping (HTCP) algorithm to identify patients at risk of developing type 2 diabetes. When a patient visits multiple healthcare centers during a study period, his/her data is stored in different EMRs and is called fragmented. In such cases, using HTPC algorithm can lead to improper classification. An experiment performed in a rural setting showed that using data from two healthcare centers instead of one decreased the false negative rate from 32.9% to 0%. In another study, researchers [51] utilized sparse logistic regression to predict type 2 diabetes risk from insurance claims data. They developed a model that outperformed the traditional risk prediction methods for large data sets and data sets with missing value cases by increasing the AUC value from 0.75 to 0.80. The dataset contained more than 500 features including demography, specific medical conditions, and comorbidity. And in another study, researchers [53] developed prediction and risk diagnosis model using a hybrid system with SVM. Using features like blood pressure, fasting blood sugar, two-hour post-glucose tolerance, cholesterol level along with other demographic and anthropometric features, the SVM algorithm was able to predict diabetes risk with 97% accuracy. One reason for achieving high accuracy compared to the study using insurance claims data [51] is the structured nature of the data which came from a cross-sectional survey on diabetes.

Different statistical and machine learning algorithms are available for classification purposes. Researchers [50] compared the performance of two statistical method (LR and Fisher linear discriminant analysis) and four machine learning algorithms (SVM (using radial basis function kernel), ANN, Random Forest, and Fuzzy C-mean) for predicting diabetes diagnosis. Ten features (age, gender, BMI, waist circumference, smoking, job, hypertension, residential region (rural/urban), physical activity, and family history of diabetes) were used to test the classification performance (diabetes or no diabetes). Parameters for ANN and SVM were optimized through Greedy search. SVM showed best performance in all performance measures. SVM was at least 5% more accurate than other classification techniques. Statistical methods performed similar to the other machine learning algorithms. This study was limited by a low prevalence of diabetes in the dataset, however, which can cause poor classification performance. Researchers [47] also proposed a novel pattern recognition algorithm by using convolutional nonnegative matrix factorization. They considered a patient as an entity and each of patients’ visit to the doctor, prescriptions, test result, and diagnosis are considered as an event over time. Finding such patterns can be helpful to group similar patients, identify their treatment pathway as well as patient management. Though they did not compare the pattern recognition accuracy with existing methods like single value decomposition (SVD), the matrix-like representation makes it intuitive.

#### 4.1.3. Cancer

Cancer is another major threat to public health [83]. Machine learning has been applied to cancer patients to predict survival, and diagnosis. We reviewed five studies that applied healthcare analytics to cancer.

Despite many advances in treatment, accurate prediction of survival in patients with cancer remains challenging considering the heterogeneity of cancer complexity, treatment options, and patient population. Survival of prostate cancer patients has been predicted using a classification model [54]. The model used a public database-SEER (Surveillance, Epidemiology, and End Result) and applied a stratified ten-fold sampling approach. Survival prediction among prostate cancer patients was made using DT, ANN and SVM algorithm. SVM outperformed other algorithms with 92.85% classification accuracy wherein DT and ANN achieved 90% and 91.07% accuracy respectively. This same database has been used to predict survival of lung cancer patients [56]. After preprocessing the 11 features available in the data set, authors identified two features (1. removed and examined regional lymph node count and 2. malignant/in-situ tumor count) which had the strongest predictive power. They used several supervised classification methods on the preprocessed data; ensemble voting of five decision tree based classifiers and meta-classifiers (J48 DT, RF, LogitBoost, Random Subspace, and Alternating DT) provided the best performance—74% for 6 months, 75% for 9 months, 77% for 1 year, 86% for 2 years, and 92% for 5 years survival. Using this technique, they developed an online lung cancer outcome calculator to estimate the risk of mortality after 6 months, 9 months, 1 year, 2 years and 5 years of diagnosis.

In addition to predicting survival, machine learning techniques have also been used to identify patients with cancer. Among patients with breast cancer, researchers [38] have proposed a new hybrid algorithm to classify breast cancer patient from patients who do not have breast cancer. They used correlation and regression to select the significant features at the first stage. Then, at the second stage, they used discrete Particle Swarm Optimization (PSO) to classify the data. This hybrid algorithm was applied to Wisconsin Breast Cancer Data set available at UCI machine learning repository. It achieved better accuracy (98.71%) compared to a genetic algorithm (GA) (96.14%) [84] and another PSO-based algorithm (93.4%) [85].

Machine learning has also been used to identify the nature of cancer (benign or malignant) and to understand demographics related to cancer. Among patients with breast cancer, researchers [42] applied the Fuzzy standard additive model (SAM) with GA (discussed earlier in relation to CVD)-predicting the nature of breast cancer (benign or malignant). They used a UCI machine learning repository which was capable of classifying uncertain and high dimensional data with greater accuracy (by 1–2%). Researchers have also used big data [55] to create a visualization tool to provide a dynamic view of cancer statistics (e.g., trend, association with other diseases), and how they are associated with different demographic variables (e.g., age, sex) and other diseases (e.g., diabetes, kidney infection). Use of data mining provided a better understanding of cancer patients both at demographic and outcome level which in terms provides an opportunity of early identification and intervention.

#### 4.1.4. Emergency Care

The Emergency department (ED) is the primary route to hospital admission [58]. In 2011, 20% of US population had at least one or more visits to the ED [86]. EDs are experiencing significant financial pressure to increase efficiency and throughput of patients. Discrete event simulation (i.e., modeling system operations with sequence of isolated events) is a useful tool to understand and improve ED operations by simulating the behavior and performance of EDs. Certain features of the ED (e.g., different types of patients, treatments, urgency, and uncertainty) can complicate simulation. One way to handle the complexity is to group the patients according to required treatment. Previously, the “casemix” principle, which was developed by expert clinicians to groups of similar patients in case-specific settings (e.g., telemetry or nephrology units), was used, but it has limitations in the ED setting [58]. Researchers applied [58] data mining (clustering) to the ED setting to group the patients based on treatment pattern (e.g., full ward test, head injury observation, ECG, blood glucose, CT scan, X-ray). The clustering model was verified and validated by ED clinicians. These grouping data were then used in discrete event simulation to understand and improve ED operations (mainly length of stay) and process flows for each group.

Chest pain admissions to the ED have also been examined using decision-making framework. Researchers [57] proposed a three stage decision-making framework for classifying severity of chest pain as: AMI, angina pectoris, or other. At the first stage, lab tests and diagnoses were collected and the association between them were extracted. In the second stage, experts developed association rules between lab tests diagnosis to help physicians make quick diagnostic decisions based diagnostic tests and avoid further unnecessary lab tests. In the third stage, authors developed a classification tree to classify the chest pain diagnosis based on selected lab test, diagnosis and medical record. This hybrid model was applied to the emergency department at one hospital. They developed the classification system using 327 association rules to selected lab tests using C5.0, Neural Network (NN) and SVM. C5.0 algorithm achieved 94.18% accuracy whereas NN and SVM achieved 88.89% and 85.19% accuracy respectively.

#### 4.1.5. Intensive Care

Intensive care units cater to patients with severe and life-threatening illness and injury which require constant, close monitoring and support to ensure normal bodily function. Death is a much more common event in an ICU compared to a general medical unit—one study showed that 22.4% of total death in hospitals occurred in the ICU [87]. Survival predictions and identification of important factors related to mortality can help healthcare providers plan care. We identified two papers [59,60] that developed prediction models for ICU mortality rate prediction. Using a large amount of ICU patient data (specifically from the first 24 h of the stay) collected from University of Kentucky Hospital from 1998 to 2007 (38,474 admissions), one group of researchers identified 15 out of 40 significant features using Pearson’s Chi-square test (for categorical variables) and Student-t test (for continuous variable) [59]. The mortality rate was predicted by DT, ANN, SVM and APACHE III, a logistic regression based approach. Compared to the other methods applied, DT’s AUC value was higher by 0.02. The study was limited, however, by only considering the first 24 h of admission to the ICU, which may not be enough to make prediction on mortality rate. Another team of researchers [60] applied a similarity metric to predict 30-day mortality prediction in 17,152 ICU admissions data extracted from MIMIC-II database [88]. Their analysis concluded that a large group of similar patient data (e.g., vital sign, laboratory test result) instead of all patient data would lead to slightly better prediction accuracy. The logistic regression model for mortality prediction achieved 0.83 AUC value when 5000 similar patients were used for training but, its performance declined to 0.81 AUC when all the available patient data were used.

#### 4.1.6. Other Applications

In addition to CVD, diabetes, cancer, emergency care, and ICU care, data mining has been applied to various clinical decision-making problems like pressure ulcer risk prediction, general problem lists, and personalized medical care. To predict pressure ulcer formation (localized skin and tissue damage because of shear, friction, pressure or any combination of these factors), researchers [62] developed two classification-based predictive models. One included all 14 features (including age, sex, course, Anesthesia, body position during operation, and skin status) and another, reduced model, including significant features only (5 in DT model, 7 in SVM, LR and Mahalanobis Taguchi System model). Mahalanobis Taguchi System (MTS), SVM, DT, and LR were used for both classification and feature selection (in the second model only) purposes. LR and SVM performed slightly better when all the features were included, but MTS achieved better sensitivity and specificity in the reduced model (+10% to +15%). These machine learning techniques can provide better assistance in pressure ulcer risk prediction than the traditional Norton and Braden medical scale [62]. Though the study provides the advantages of using data mining algorithms, the data set used here was imbalanced as it only had 8 cases of pressure ulcer in 168 patients. Also among patients with pressure ulcers, another team of researchers [63] recommended a data mining based alternative to the Braden scale for prediction. They applied data mining algorithms to four years of longitudinal patient data to identify the most important factors related to pressure ulcer prediction (i.e., days of stay in the hospital, serum albumin, and age). In terms of C-statistics, RF (0.83) provided highest predictive accuracy over DT (0.63), LR (0.82), and multivariate adaptive regression splines (0.78).

For data mining algorithms, which often show poor performance with imbalanced (i.e., low occurrence of one class compared to other classes) data, researchers [70] developed a sub-sampling technique. They designed two experiments, one considered sub-sampling technique and another one did not. For a highly imbalanced data set, Random Forest (RF), SVM, and Bagging and Boosting achieved better classification accuracy with this sub-sampling technique in classifying eight diseases (male genital disease, testis cancer, encephalitis, aneurysm, breast cancer, peripheral atherosclerosis, and diabetes mellitus) that had less than 5% occurrences in the National Inpatient Sample (NIS) data of Healthcare Cost and Utilization Project (HCUP). Surprisingly, possibly due to balancing the dataset through sub-sampling, RF slightly outperformed (+0.01 AUC) the other two methods.

The patient problem list is a vital component of clinical medicine. It enables decision support and quality measurement. But, it is often incomplete. Researchers have [64] suggested that a complete list of problems leads to better quality treatment in terms of final outcome [64]. Complete problem lists enable clinicians to get a better understanding of the issue and influence diagnostic reasoning. One group of researchers proposed a data mining model to find an association between patient problems and prescribed medications and laboratory tests which can act as a support to clinical decision-making [64]. Currently, domain experts spend a large amount of time for this purpose but, association rule mining can save both time and other resources. Additionally, consideration of unstructured data like doctor’s and/or nurse’s written comments and notes can provide additional information. These association rules can aid clinicians in preventing errors in diagnosis and reduce treatment complexity. For example, a set of problems and medications can co-occur frequently. If a clinician has knowledge about this relation, he/she can prescribe similar medications when faced with a similar set of problems. One group of researchers [61] developed an approach which achieved 90% accuracy in finding association between medications and problems, and 55% accuracy between laboratory tests and problems. Among outpatients diagnosed with respiratory infection, 92.79% were treated with drugs. Physicians could choose any of the 100,013 drugs available in the inventory. Moreover, in an attempt to examine the treatment plan patterns, they identified the 78 most commonly used drugs which could be prescribed, regardless of patient’s complaints and demography. The classification model used to identify the most common drugs achieved 74.73% accuracy and most importantly found variables like age, race, gender, and complaints of patients were insignificant.

Personalized medicine—tailored treatment based on a patient’s predicted response or risk of disease—is another venue for data mining algorithms. One group of researchers [66] used a big data framework to create personalized care system. One patient’s medical history is compared with other available patient data. Based on that comparison, possibility of a disease of an individual was calculated. All the possible diseases were ranked from high risk to low risk diseases. This approach is very similar to how online giants Netflix and Amazon suggest movies and books to the customer [66]. Another group of researchers [67] used the Electronic Patient Records (EPR), which contains structured data (e.g., disease code) and unstructured data (e.g., notes and comments made by doctors and nurses at different stages of treatment) to develop personalized care. From the unstructured text data, the researchers extracted clinical terms and mapped them to an ontology. Using this mapped codes and existing structured data (disease code), they created a phenotypic profile for each patient. The patients were divided into different clusters (with 87.78% precision) based on the similarity of their phenotypic profile. Correlation of diseases were captured by counting the occurrences of two or more diseases in patient phenotype. Then, the protein/gene structure associated with the diseases was identified and a protein network was created. From the sharing of specific protein structure by the diseases, correlation was identified.

Among patients with asthma, researchers [65] used environmental and patient physiological data to develop a prediction model for asthma attack to give doctors and patients a chance for prevention. They used data from a home-care institute where patients input their physical condition online; and environmental data (air pollutant and weather data). Their data mining model involved feature selection through sequential pattern mining and risk prediction using DT and association rule mining. This model can make asthma attack risk prediction with 86.89% accuracy. Real implementation showed that patients found risk prediction helpful to avoid severe asthma attacks.

Among patients with Parkinson’s disease, researchers [73] introduced a comprehensive end-to-end protocol for complex and heterogeneous data characterization, manipulation, processing, cleaning, analysis and validation. Specifically, the researchers used a Synthetic Minority Over-sampling Technique (SMOTE) to rebalance the data set. Rebalancing the dataset using SMOTE improved SVM’s classification accuracy from 76% to 96% and AdaBoost’s classification accuracy from 96% to 99%. Moreover, the study found that traditional statistical classification approaches (e.g., generalized linear model) failed to generate reliable predictions but machine learning-based classification methods performed very well in terms of predictive precision and reliability.

Among patients with kidney disease, researchers [71] developed a prediction model to forecast survival. Data collected from four facilities of University of Iowa Hospital and Clinics contains 188 patients with over 707 visits and features like blood pressure measures, demographic variables, and dialysis solution contents. Data was transformed using functional relation (i.e., the similarity between two or more features when two features have same values for a set of patients, they are combined to form a single feature) between the features. The data set was randomly divided into eight sub-sets. Sixteen classification rules were generated for the eight sub-sets using two classification algorithms—Rough Set (RS) and DT. Classes represented survival beyond three years, less than three years and undetermined. To make predictions, each classification rule (out of 16) had one vote and the majority vote decided the final predictive class. Transformed data increased predictive accuracy by 11% than raw data and DT (67% accuracy) performed better than RS (56% accuracy). The researchers suggested that this type of predictive analysis can be helpful in personalized treatment selection, resource allocation for patients, and designing clinical study. Among patients on kidney dialysis, another group of researchers [74] applied temporal pattern mining to predict hospitalization using biochemical data. Their result showed that amount of albumin—a type of protein float in blood—is the most important predictor of hospitalization due to kidney disease.

Among patients over 50 years of age, researchers [75] developed a data mining model to predict five years mortality using the EHR of 7463 patients. They used Ensemble Rotating Forest algorithm with alternating decision tree to classify the patients into two classes of life expectancy: (1) less than five years and (2) equal or greater than five years. Age, comorbidity count, previous record of hospitalization record, and blood urea nitrogen were a few of the significant features selected by correlation feature selection along with greedy stepwise search method. Accuracy achieved by this approach (AUC 0.86) was greater than the standard modified Charlson Index (AUC 0.81) and modified Walter Index (AUC 0.78). Their study showed that age, hospitalization prior the visit, and highest blood urea nitrogen were the most important factors for predicting five years morbidity. This five-year morbidity prediction model can be very helpful to optimally use resources like cancer screening for those patients who are more likely to be benefit from the resources.

Another group of researchers [76] addressed the limitations of existing software technology for disease diagnosis and prognosis, such as inability to handle data stream (DT), impractical for complex and large systems (Bayesian Network), exhaustive training process (NN). To overcome these restriction, authors proposed a decision tree based algorithm called “Very Fast Decision Tree (VFDT)”. Comparison with a similar system developed by IBM showed that VFDT utilizes lesser amount of system resources and it can perform real time classification.

Researchers have also used data mining to optimize the glaucoma diagnosis process [68]. Traditional approaches including Optical Coherence Tomography, Scanning Laser Polarimetry (SLP), and Heidelberg Retina Tomography (HRT) scanning methods are costly. This group used Fundus image data which is less costly and classified patient as either normal or glaucoma patient using SVM classifier. Before classification, authors selected significant features by using Higher Order Spectra (HOS) and Discrete Wavelet Transform (DWT) method combined and separately. Several kernel functions for SVM—all delivering similar levels of accuracy—were applied. Their approach produced 95% accuracy in glaucoma prediction. For diagnostic evaluation of chest imaging for suspicion for malignancy, researchers [69] designed trigger criteria to identify potential follow-up delays. The developed trigger predicted the patients who didn’t require follow-up evaluation. The analysis of the experiment result indicated that the algorithm to identify patients’ delays in follow-up of abnormal imaging is effective with 99% sensitivity and 38% specificity.

Data mining has also been applied to [72] compare three metrics to identify health care associated infections—Catheter Associated Bloodstream Infections, Catheter Associated Urinary Tract Infections and Ventilator Associated Pneumonia. Researchers compared traditional surveillance using National Healthcare Safety Network methodology to data mining using MedMined Data Mining Surveillance (CareFusion Corporation, San Diego, CA, USA), and administrative coding using ICD-9-CM. Traditional surveillance proved to be superior than data mining in terms of sensitivity, positive predictive value and rate estimation.

Data mining has been used in 38 studies of clinical decision-making CVD (7 articles), diabetes (seven articles), cancer (five articles), emergency care (two articles), intensive care (two articles), and other applications (16 articles). Most of the studies developed predictive models to facilitate decision-making and some developed decision support system or tools. Authors often tested their models with multiple algorithms; SVM was at the top of that list and often outperformed other algorithms. However, 15 [38,40,42,45,47,51,54,56,58,60,61,66,73,74,76] of the studies did not incorporate expert opinion from doctors, clinician, or appropriate healthcare personals in building models and interpreting results (see the study characteristics in Supplementary Materials Table S3). We also noted that there is an absence of follow-up studies on the predictive models, and specifically, how the models performed in dynamic decision-making situations, if doctors and healthcare professionals comfortable in using these predictive models, and what are the challenges in implementing the models if any exist? Existing literature does not focus on these salient issues.

### 4.2. Healthcare Administration

Data mining was applied to administrative purposes in healthcare in 32% (29 articles) of the articles reviewed. Researchers have applied data mining to: data warehousing and cloud computing; quality improvement; cost reduction; resource utilization; patient management; and other areas. Table 6 provides a list of these articles with major focus areas, problems analyzed and the data source.

#### 4.2.1. Data Warehousing and Cloud Computing

Data warehousing [90] and cloud computing are used to securely and cost-effectively store the growing volume of electronic patient data [1] and to improve hospital outcomes including readmissions. To identify cause of readmission, researchers [89] developed an open source software—Analytic Information Warehouse (AIW). Users can design a virtual data model (VDM) using this software. Required data to test the model can be extracted in terms of a temporal ontology from the data warehouse and analysis can be performed using any standard analyzing tool. Another group of researchers took a similar approach to develop a Clinical Data Warehouse (CDW) for traditional Chinese medicine (TCM). The warehouse contains clinical information (e.g., symptoms, disease, and treatment) for 20,000 inpatients and 20,000 outpatients. Data was collected in a structured way using pre-specified ontology in electronic form. CDW provides an interface for online data mining, online analytical processing (OLAP) and network analysis to discover knowledge and provide clinical decision support. Using these tools, classification, association and network analysis between symptoms, diseases and medications (i.e., herbs) can be performed.

Apart from clinical purposes, data warehouses can be used for research, training, education, and quality control purposes. Such a data repository was created using the basic idea of Google search engine [92]. Users can pull the radiology report files by searching keywords like a simple google search following the predefined patient privacy protocol. Another data repository was created as a part of collaborative study between IBM and University of Virginia and its partner, Virginia Commonwealth University Health System was created [93]. The repository contains 667,000 patient record with 208 attributes. HealthMiner—a data mining package for healthcare created by IBM—was used to perform unsupervised analysis like finding associations, pattern and knowledge discovery. This study also showed the research benefits of this type of large data repository. Researchers [91] proposed a framework based on cloud computing and big data to unify data collected from different sources like public databases and personal health devices. The architecture was divided into 3 layers. The first layer unified heterogeneous data from different sources, the second layer provided storage support and facilitated data processing and analytics access, and the third layer provided result of analysis and platform for professionals to develop analytical tools. Some researchers [94] used mobile devices to collect personal health data. Users took part in a survey on their mobile devices and got a diagnosis report based on their health parameters input in the survey. Each survey data were saved in a cloud-based interface for effective storage and management. From user input stored in cloud, interactive geo-spatial maps were developed to provide effective data visualization facility.

#### 4.2.2. Healthcare Cost, Quality and Resource Utilization

Ten articles applied data mining to cost reduction, quality improvement and resource utilization issues. One group of researchers predicted healthcare costs using an algorithmic approach [96]. They used medical claim data of 800,000 people collected by an insurance company over the period of 2004–2007. The data included diagnoses, procedures, and drugs. They used classification and clustering algorithms and found that these data mining algorithms improve the absolute prediction error more than 16%. Two prediction models were developed, one using both cost and medical information and the other used only cost information. Both models had similar accuracy on predicting healthcare costs but performed better than traditional regression methods. The study also showed that including medical information does not improve cost prediction accuracy. Risk-adjusted health care cost predictions, with diagnostic groups and demographic variables as inputs, have also been assessed using regression tree boosting [100]. Boosted regression tree and main effects linear models were used and fitted to predict current (2001) and prospective (2002) total health care costs per patient. The authors concluded that the combination of regression tree boosting and a diagnostic grouping scheme are a competitive alternative to commonly used risk-adjustment systems.

A sizable amount ($37.6 billion) of healthcare costs is attributable to medical errors, 45% of which stems from preventable errors [95]. To aid in physician decision-making and reduce medical errors, researchers [95] proposed a data mining-based framework-Sequential Clustering Algorithm. They identified patterns of treatment plans, tests, medication types and dosages prescribed for specific diseases, and other services provided to treat a patient throughout his/her stay in the hospital. The proposed framework was based on cloud computing so that the knowledge extracted from the data could be shared among hospitals without sharing the actual record. They proposed to share models using Virtual Machine (VM) images to facilitate collaboration among international institutions and prevent the threat of data leakage. This model was implemented in two hospitals, one in Taiwan and another in Mongolia. To identify best practices for specific diseases and prevent medical errors, another group of researchers [101] proposed a decision support system using information extraction from online documents through text and data mining. They focused on evidence based management, quality control, and best practice recommendations for medical prescriptions.

Length of Stay (LOS) is another important indicator of cost and quality of care. Accurate prediction of LOS can lead to efficient management of hospital beds and resources. To predict LOS for CAD patients, researchers [98] compared multiple models—SVM, ANN, DT and an ensemble algorithm, combing SVM, C5.0, and ANN. Ensemble algorithm and SVM produced highest accuracy, 95.9% and 96.4% respectively. In contrast, ANN was least accurate with 53.9% accuracy wherein DT achieved 83.5% accuracy. Anticoagulant drugs, nitrate drugs, and diagnosis were the top three predictors along with diastolic blood pressure, marital status, sex, presence of comorbidity, and insurance status.

To predict healthcare quality, researchers [104] used sentiment analysis (computationally categorizing opinions into categories like positive, negative and neutral) on patients’ online comments about their experience. They found above 80% agreement between sentiment analysis from online forums and traditional paper based surveys on quality prediction (e.g., cleanliness, good behavior, recommendation). Proposed approach can be an inexpensive alternative to traditional surveys and reports to measure healthcare quality.

Identification of influential factors in insurance coverage using data mining can aid insurance providers and regulators to design targeted service, additional service or proper allocation of resources to increase coverage rates. To develop a classification model to identify health insurance coverage, researchers [103] used data mining techniques. Based on 23 socio-economic, lifestyle and demographic factors, they developed a classification model with two classes, Insured and uninsured. The model was solved by ANN and DT. ANN provided 4% more accuracy than DT in predicting health insurance coverage. Among the factors, income, employment status, education, and marital status were the most important predictive factors of insurance coverage.

Among patients with lung cancer, researchers [97] investigated healthcare resource utilization (i.e., the number of visits to the medical oncologists) characteristics. They used DT, ANN and LR separately and an ensemble algorithm combining DT and ANN which resulted in the greatest accuracy (60% predictive accuracy). DT was employed to identify the important predictive features (among demographics, diagnosis, and other medical information) and ANN for classification. Data mining revealed that the utilization of healthcare resources by lung cancer patients is “supply-sensitive and patient sensitive” where supply represents availability of resources in certain region and patient represents patient preference and comorbidity. A resource allocation monitoring model for better management of primary healthcare network has also been developed [99]. Researchers considered the primary-care network as a collection of hierarchically connected modules given that patients could visit multiple physicians and physicians could have multiple care location, which is an indication of imbalanced resource distribution (e.g., number of physicians, care locations). The first level of the hierarchy consisted of three modules: health activities, population, and health resources. The second level monitored the healthcare provider availability and dispersion. The third level considered the actual visits, physicians and their availability, accessibility, and unlisted (i.e., without any assigned physician) patients. The top level of this network conducted an overall assessment of the network and made allocation accordingly. This hierarchical model was developed for a specific region in Slovenia, however, it could be easily adapted for any other region.

Overuse of screening and tests by physicians also contributes to inefficiencies and excess costs [102]. Current practice in pathology diagnosis is limited by disease focus. As an alternative to disease based system, researchers [102] used data mining in cooperation with case-based reasoning to develop an evidence based decision support system to decrease the use of unnecessary tests and reduce costs.

#### 4.2.3. Patient Management

Patient management involves activities related to efficient scheduling and providing care to patients during their stay in a healthcare institute. Researchers [105] developed an efficient scheduling system for a rural free clinic in the United States. They proposed a hybrid system where data mining was used to classify the patients and association rule mining was used to assign a “no-show” probability. Results obtained from data mining were used to simulate and evaluate different scheduling techniques. On the other hand, these schedules could be divided into visits with administrative purposes and medical purposes. Researchers [108] suggested that patients who visit the health center for administrative purposes take less time than the patients with medical reasons. They proposed a predictive model to forecast the number of visits for administrative purposes. Their model improved the scheduling system with time saving of 21.73% (660,538 min). In contrast to administrative information/task seeking patients, some patients come for medical care very frequently and consume a large percentage of clinical workload [107]. Identifying the risk factors for frequent visit to health centers can help in reducing cost and resource utilization. A study among 85 working age “frequent attenders” identified the primary risk factors using Bayesian classification technique. The risk factors are, “high body mass index, alcohol abstinence, irritable bowel syndrome, low patient satisfaction, and fear of death” [107].

Improving publicly reported patient safety outcomes is also critical to healthcare institutions. Falls are one such outcome and are the most common and costly source of injury during hospitalization [110]. Researchers [109] analyzed the important factors related to patient falls during hospitalization. First, the authors selected significant features by Chi-square test (10 features out of 72 fall related variables were selected) and then applied ANN to develop a predictive model which achieves 0.77 AUC value. Stepwise logistic regression achieved 0.42 AUC value with 3 important variables. Both models showed that the fall assessment by nurses and use of anti-psychotic medication are associated with a lower risk of falls, and the use of diuretics is associated with an increased risk of falls. Another group of researchers [110] used fall related injury data to validate the structured information in EMR from clinical notes with the help of text mining. A group of nurses manually reviewed the electronic records to separate the correct documents from the erroneous ones which was considered as the basis of comparison. Authors employed both supervised (using a portion of manually labeled files as training set) and unsupervised technique (without considering the file labels) to classify and cluster the records. The unsupervised technique failed to separate the fare documents from the erroneous ones, wherein supervised technique performed better with 86% of fare documents in one cluster. This method can be applicable to semi-automate the EMR entry system.

#### 4.2.4. Other Applications

Data mining has beed applied [111] to investigate the relationship between physician’s training at specific schools, procedures performed, and costs of the procedure. Researchers explored this relationship at three level: (1) they explored the distribution of procedures performed; (2) the relationship between procedures performed by physician and their alma mater—the institute that a doctor attended or got his/her degree from; and (3) geographic distribution of amount billed and payment received. This study suggested that medical school training does relate to practice in terms of procedures performed and bill charged. Patients can also provide useful information about physicians and their performance. Another group of researchers [112] used topic modeling algorithm—Latent Dirichlet Allocation (LDA)—to understand patients’ review of physicians and their concerns.

Data mining has also been applied [115] to analyze the information seeking behavior of health care professionals, and to assess the feasibility of measuring drug safety alert response from the usage logs of online medical information resources. Researchers analyzed two years of user log-in data in UpToDate website to measure the volume of searches associated with medical conditions and the seasonal distribution of those searches. In addition, they used a large collection of online media articles and web log posts as they characterized food and drug alert through the changes in UpToDate search activity compared to the general media activity. Some researchers [113] examined changes of key performance indicators (KPIs) and clinical workload indicators in Greek National Health System (NHS) hospitals with the help of data mining. They found significant changes in KPIs when necessary adjustments (e.g., workload) were made according to the diagnostic related group. The results remained for general hospitals like cancer hospitals, cardiac surgery as well as small health centers and regional hospitals. Their findings suggested that the assessment methodology of Greek NHS hospitals should be re-evaluated in order to identify the weaknesses in the system, and improve overall performance. And in home healthcare, another group of researchers [116] reviewed why traditional statistical analysis fails to evaluate the performance of home healthcare agencies. The authors proposed to use data mining to identify the drivers of home healthcare service among patients with heart failure, hip replacement, and chronic obstructive pulmonary disease using length of stay and discharge destination.

The relationship between epidemiological and genetic evidence and post market medical device performance has been evaluated using HCUPNet data [114]. This feasibility study explored the potential of using publicly accessible data for identifying genetic evidence (e.g., comorbidity of genetic factors like race, sex, body structure, and pneumothorax or fibrosis) related to devices. It focused on the ventilation-associated iatrogenic pneumothorax outcome in discharge of mechanical ventilation and continuous positive airway pressure (CPAP). The results demonstrated that genetic evidence-based epidemiologic analysis could lead to both cost and time efficient identification of predictive features. The literature of data mining applications in healthcare administration encompasses efficient patient management, healthcare cost reduction, quality of care, and data warehousing to facilitate analytics. We identified four studies that used cloud-based computing and analytical platforms. Most of the research proposed promising ideas, however, they do not provide the results and/or challenges during and after implementation. An ideal example of implementation could be the study of efficient appointment scheduling of patients [108].

### 4.3. Healthcare Privacy and Fraud Detection

Health data privacy and medical fraud are issues of prominent importance [118]. We reviewed four articles—displayed and described in Table 7—that discussed healthcare privacy and fraud detection.

The challenges of privacy protection have been addressed by a group of researchers [122] who proposed a new anonymization algorithm for both distributed and centralized anonymization. Their proposed model performed better than K-anonymization model in terms of retaining data utility without losing much data privacy (for K = 20, the discernibility ratio—a normalized measure of data quality—of the proposed approach and traditional K-anonymization method were 0.1 and 0.4 respectively). Moreover, their proposed algorithm could handle large scale, high dimensional datasets. To address the limitations of today’s healthcare information systems—EHR data systems limited by lack of inter-operability, data size, and security—a mobile cloud computing-based big data framework has been proposed [119]. This novel cloud-based framework proposed storing EHR data from different healthcare providers in an Internet provider’s facility, offering providers and patients different levels of access and authority. Security would be ensured by using encryption algorithms, one-time passwords, or 2-factor authentication. Big data analytics would be handled using Google big query or MapReduce software. This framework could reduce cost, increase efficiency, and ensure security compared to the traditional technique which uses de-identification or anonymization technique. This traditional technique leaves healthcare data vulnerable to re-identification. In a case study, researchers demonstrated that hackers can make association between small pieces of information and can identify patients [120]. The case study made use of personal information provided in two Medicare social networking sites, MedHelp and Mp and Th1 to identify an individual.

Detection of fraud and abuse (i.e., suspicious care activity, intentional misrepresentation of information, and unnecessary repetitive visits) uses big data analytics. Using gynecological hospital data, researchers [121] developed a framework from two domain experts manually identifying features of fraudulent cases from a data pool of treatment plans doctors frequently follow. They applied this framework to Bureau of National Health Insurance (BNHI) data from Taiwan; their proposed framework detected 69% of the fraudulent cases, which improved the existing model that detected 63% of the fraudulent cases.

In summary, patient data privacy and fraud detection are of major concern given increasing use of social media and people’s tendency to put personal information on social media. Existing data anonymization or de-identification techniques can become less effective if they are not designed considering the fact that a large portion of our personal information is now available on social media.

### 4.4. Mental Health

Mental illness is a global and national concern [123]. According to the National Survey on Drug Use and Health (NSDUH) data from 2010 to 2012, 52.2% of U.S. population had either mental illness, or substance abuse/dependence [124]. Additionally, nearly 30 million people in the U.S. suffer from anxiety disorders [125]. Table 8 summarizes the four articles we reviewed that apply data mining in analyzing, diagnosing, and treating mental health issues.

To classify developmental delays of children based on illness, researchers [126] examined the association between illness diagnosis and delays by building a decision tree and finding association between cognitive, language, motor, and social emotional developmental delays. This study has implications for healthcare professionals to identify and intervene on delays at an early stage. To assist physicians in monitoring anxiety disorder, another group of researchers [125] developed a data mining based personalized treatment. The researchers used Context Awareness Information including static (personal information like, age, sex, family status etc.) and dynamic (stress, environmental, and symptoms context) information to build static and dynamic user models. The static model contained personal information and the dynamic model contained four treatment-supportive services (i.e., lifestyle and habits pattern detection service, context and stress level pattern detection service, symptoms and stress level pattern detection service, and stress level prediction service). Relations between different dynamic parameters were identified in first three services and the last service was used for stress level prediction under different scenarios. The model was validated using data from 27 volunteers who were selected by anxiety measuring test.

To predict early diagnosis for mental disorders (e.g., insomnia, dementia), researchers developed a model detecting abnormal physical activity recorded by a wearable device [127]. They performed two experiments to compare the development of a reference model using historical user physical movement data. In the first experiment, users wore the watch for one day and based on that day, a reference behavior model was developed. After 22 days, the same user used it again for a day and abnormality was detected if the user’s activities were significantly different from the reference model. In the second experiment, users used the watch regularly for one month. Abnormality was detected with a fuzzy valuation function and validated with user’s reported activity level. In both experiments, users manually reported their activity level, which was used as a validating point, only two out of 26 abnormal events were undetected. Through these two experiments, the researchers claimed that their model could be useful for both online and offline abnormal behavior detection as the model was able to detect 92% of the unusual events.

To classify schizophrenia, another study [128] used free speech (transcribed text) written or verbalized by psychiatric patients. In a pool of patients with schizophrenia and control subjects, using supervised algorithms (SVM and DT), they discriminated between patients with schizophrenia and normal control patients. SVM achieved 77% classification accuracy whereas DT achieved 78% accuracy. When they added patients with mania to the pool, they were unable to differentiate patients with schizophrenia.

Use of data analytics in diagnosing, analyzing, or treating mental health patients is quite different than applying analytics to predict cancer or diabetes. Context of data (static, dynamic, or unobservable environment) seemed more important than volume in this case [125], however, this is not always adopted in literature. A model without situational awareness (a context independent model) may lose predictive accuracy due to the confounding effect of surrounding environment [129].

### 4.5. Public Health

Seven articles addressed issues that were not limited to any specific disease or a demographic group, which we classified as public health problems. Table 9 contains the list of papers considering public health problems with data sources.

To make data mining accessible to non-expert users, specifically public health decision makers who manage public cancer treatment programs in Brazil, researchers [134] developed a framework for an automated data mining system. This system performed a descriptive analysis (i.e., identifying relationships between demography, expenditure, and tumor or cancer type) for public decision makers with little or no technical knowledge. The automation process was done by creating pre-processed database, ontology, analytical platform and user interface.

Analysis of disease outbreaks has also applied data analytics. [131,133] Influenza, a highly contagious disease, is associated with seasonal outbreaks. The ability to predict peak outbreaks in advance would allow for anticipatory public health planning and interventions to lessen the effect of the outbreaks. To predict peak influenza visits to U.S. military health centers, researchers [131] developed a method to create models using environmental and epidemiological data. They compared six classification algorithms—One-Classifier 1, One-Classifier 2 [137], a fusion of the One-Classifiers, DT, RF, and SVM. Among them, One-Classifier 1 was the most efficient with F-score 0.672 and SVM was second best with F-score 0.652. To examine the factors that drive public and professional search patterns for infectious disease outbreaks another group of researchers [133] used online behavior records and media coverage. They identified distinct factors that drive professional and layperson search patterns with implications for tailored messaging during outbreaks and emergencies for public health agencies.

To store and integrate multidimensional and heterogeneous data (e.g., diabetes, food, nutrients) applied to diabetes management, but generalizable to other diseases researchers [130] proposed an intelligent information management framework. Their proposed methodology is a robust back-end application for web-based patient-doctor consultation and e-Health care management systems with implications for cost savings.

A real-time medical emergency response system using the Internet of Things (networking of devices to facilitate data flow) based body area networks (BANs)—a wireless network of wearable computing devices was proposed by researchers [136]. The system consists of “Intelligent Building”—a data analysis model which processes the data collected from the sensors for analysis and decision. Though the author claims that the proposed system had the capability of efficiently processing wireless BAN data from millions of users to provide real-time response for emergencies, they did not provide any comparison with the state-of-the-art methods.

Decision support tools for regional health institutes in Slovenia [135] have been developed using descriptive data mining methods and visualization techniques. These visualization methods could analyze resource availability, utilization and aid to assist in future planning of public health service.

To build better customer relations management at an Iranian hospital, researchers [132] applied data mining techniques on demographic and transactions information. The authors extended the traditional Recency, Frequency, and Monetary (RFM) model by adapting a new parameter “Length” to estimate the customer life time value (CLV) of each patient. Patients were separated into classes according to estimated CLV with a combination of clustering and classification algorithms. Both DT and ANN performed similarly in classification with approximately 90% accuracy. This type of stratification of patient groups with CLV values would help hospitals to introduce new marketing strategies to attract new customers and retain existing ones.

The application of data mining to public health decision-making has become increasingly common. Researchers utilized data mining to design healthcare programs and emergency response, to identify resource utilization, patient satisfaction as well as to develop automated analytics tool for non-expert users. Continuation of this effort could lead to a patient-centered, robust healthcare system.

### 4.6. Pharmacovigilance

Pharmacovigilance involves post-marketing monitoring and detection of adverse drug reactions (ADRs) to ensure patient safety [138]. The estimated annual social cost of ADR events exceeds one billion dollars, making it an important part of healthcare system [139]. Characteristics of the nine papers addressing pharmacovigilance are displayed in Table 10.

Researchers considered muscular and renal AEs caused by pravastatin, simvastatin, atorvastatin, and rosuvastatin by applying data mining techniques to the FDA’s Adverse Event Reporting System (FAERS) database reports from 2004 to 2009 [143]. They found that all statins except simvastatin were associated with muscular AE; rosuvastatin had the strongest association. All statins, besides atorvastatin, were associated with acute renal failure. The criteria used to identify significant association were: proportional reporting ratio (PRR), reporting odds ratio (ROR), information component (IC), and empirical Bayes geometric mean (EBGM). In another study of AEs related to statin family, researchers used a Korean claims database [145] and showed that a relative risk-based data-mining approach successfully detected signals for rosuvastatin.

Three more studies used the FDA’s AERS report database. In an examination of ADR “hypersensitivity” to six anticancer agents [142] data mining results showed that Paclitaxel is associated with mild to lethal reaction wherein Docetaxel is associated to lethal reaction, and the other four drugs were not associated to hypersensitivity [142]. Another researcher [139] argued that AEs can be caused not only by a single drug, but also by a combination of drugs [140]. They showed that that 84% of the AERs reports contain an association between at least one drug and two AEs or two drugs and one AE. Another group [138] increased precision in detecting ADRs by considering multiple data sources together. They achieved 31% (on average) improvement in identification by using publicly available EHRs in combination with the FDA’s AERS reports.

Furthermore, dose-dependent ADRs have been identified by researchers using models developed from structured and unstructured EHR data [141]. Among the top five drugs associated with ADRs, four were found to be related to dose [141]. Pharmacovigilance activity has also been prioritized using unstructured text data in EHRs [144]. In traditional pharmacovigilance, ADRs are unknown. While looking for association between a drug and any possible ADR, it is possible to get false signals. Such false signals can be avoided if a list of possible ADRs is already known. Researchers [144] developed an ordered list of 23 ADRs which can be very helpful for future pharmacovigilance activities. To detect unexpected and rare ADRs in real-world healthcare administrative databases, another group of researchers [146] designed an algorithm—Unexpected Temporal Association Rules (UTARs)—that performs more effectively than existing techniques.

We identified one study that used data outside of adverse event reports or HER data. For early detection of ADR, one group of researchers used online forums [140]. They identified the side effect of a specific drug called “Erlotinib” used for lung cancer. Sentiment analysis—a technique of categorizing opinions—on data collected from different cancer discussion forums showed that 70% of users had a positive experience after using this drug. Users most frequently reported were acne and rash. Apart from pharmacovigilance, this type of analysis can be very helpful for the pharmaceutical companies to analyze customer feedback. Researchers can take advantage of the popularity of social media and online forums for identifying adverse events. These sources can provide signals of AEs quicker than FDA database as it takes time to update the database. By the time AE reports are available in the FDA database, there could already be significant damage to patient and society. Moreover, it can help to avoid the limitations of FDA AERS database like biased reporting and underreporting [141].

## 5. Theoretical Study

Twenty-five of the articles we reviewed focus on the theoretical aspects of the application of data mining in healthcare including designing the database framework, data collection, and management to algorithmic development. These intellectual contributions extend beyond the analytical perspective of data—descriptive, predictive or prescriptive analytics—to the sectors and problems highlighted in Table 11.

The existing theoretical literature on disease control highlighted the current state of epidemics, cancer and mental health. To help physicians make real-time decisions about patient care, one group of researchers [147] proposed a real-time EMR data mining based clinical decision support system. They emphasized the need to have an anonymized EMR database which can be explored by using a search engine similar to web search engine. In addition, they focused on designing a framework for next generation EMR-based database that can facilitate the clinical decision-making process, and is also capable of updating a central population database once patients’ recent (new) clinical records are available. Another researcher [148] forecasted future challenges in infection control that entails the importance of having timely surveillance system and prevention programs in place. To that end, they necessitate the formation, control and utilization of fully computerized patient record and data-mining-derived epidemiology. Finally, they recommended performance feedback to caregivers, wide accessibility of infection prevention tools, and access to documents like lessons learned and evidence-based best practices to strengthen the infection control, surveillance, and prevention scheme. Authors in [150] addressed the activities executed by national Institute of Mental Health (NIMH) in collaboration with other state organizations (e.g., Substance Abuse and Mental Health Service Administration (SAMSHSA), Center for Mental Health Service (CMHS) to promote optimal collection, pooling/aggregation, and use of big data to support ongoing and future researches of mental health practices. The outcome summary showcased that effective pooling/aggregation of state-level data from different sources can be used as a dashboard to set priorities to improve service qualities, measure system performance and to gain specific context-based insights that are generalizable and scalable across other systems, leading to a successful learning-based mental health care system. Another group of researchers [150] outlined the barriers and potential benefits of using big data from CancerLinQ (a quality and measurement reporting system as an initiative of the American Society of Clinical Oncology (ASCO) that collects information from EHRs of cancer patients for oncologists to improve the outcome and quality of care they provide to their patients). However, the authors also mentioned that these benefits are contingent upon the confidence of the patients, encouraging them to share their data out of the belief that their health records would be used appropriately as a knowledge base to improve the quality of the health care of others, as it is for themselves. This motivated ASCO to ensure that proper policies and procedures are in place to deal with the data quality, data security and data access, and adopt a comprehensive regulatory framework to ensure patients’ data privacy and security.

Another group of researchers [151] data quality and database management to quantify, and consequentially understand the inherent uncertainty originating from radiology reporting system. They discussed the necessity of having a structured reporting system and emphasized the use of standardize language, leading to Natural Language Processing (NLP). Furthermore, they also indicated the need for creating a Knowledge Discovery Database (KDD) which will be consistent to facilitate the data-driven and automated decision support technologies to help improving the care provided to patients based on enhanced diagnosis quality and clinical outcome. A group of authors in [152] pointed that the success derived from the current trend of big-data analytics largely depends on how better the quality of the data collected from variety of sources are ensured. Their findings imply that the data quality should be assessed across the entire lifecycle of health data by considering the errors and inaccuracies stemmed from multiple of sources, and should also quantify the impact that data collection purpose on the knowledge and insights derived from the big data analytics. For that to ensure, they recommend that enterprises who deal with healthcare big data should develop a systematic framework including custom software or data quality rule engines, leading to an effective management of specific data-quality related problems. Researchers in [155] uncovered the lack of connection between phenomenological and mechanistic models in computational biomedicines. They emphasized the importance of big data which, when successfully extracted and analyzed, followed by the combination with Virtual Physiological Human (VPH)—an initiative to encourage personalized healthcare—can afford with effective and robust medicine solutions. In order for that to happen, they mentioned some challenges (e.g., confidentiality, volume and complexity of big data; integration of bioinformatics, systems biology and phenomics data; efficient storage of partial or complete data within organization to maximize the performance of overall predictive analytics) and concluded that these need to be addressed for successful development of big data technologies in computational medicines, enabling their adoption in clinical settings. Even though big data can generate significant value in modern healthcare system, researchers in [154] stated that without a set of proper IT infrastructures, analytical and visualization tools, and interactive interfaces to represent the work flows, the insights generated from big data will not be able to reach its full potential. To overcome this, they recommended that health care organizations engaging in data sharing devise new policies to protect patients’ data against potential data breaches.

Three papers [155,156,157] considered health care policies and ethical and legal issues. One [155] outlined a national action plan to incorporate sharable and comparable nursing data beyond documentation of care into quality reporting and translational research. The plan advocates for standardized nursing terminologies, common data models, and information structures within EHRs. Another paper [157] analyzed the major policy, ethical, and legal challenges of performing predictive analytics on health care big data. Their proposed recommendations for overcoming challenges raised in the four-phase life cycle of a predictive analytics model (i.e., data acquisition, model formulation and validation, testing in real-world setting and implementation and use in broader scale) included developing a governance structure at the earliest phase of model development to guide patients and participating stakeholders across the process (from data acquisition to model implementation). They also recommended that model developers strictly comply with the federal laws and regulations in concert with human subject research and patients information privacy when using patients’ data. And another paper [156] explored four central questions regarding: (i) aspects of big-data most relevant to health care, (ii) policy implications, (iii) potential obstacles in achieving policy objectives, and (iv) availability of policy levers, particularly for policy makers to consider when developing public policy for using big data in healthcare. They discussed barriers (including ensuring transparency among patients and health care providers during data collection) to achieve policy objectives based on a recent UK policy experiment, and argued for providing real-life examples of ways in which data sharing can improve healthcare.

Three papers [158,159,160] offered examples of realistic ways such as establishing policy leadership and risk management framework combining commercial and health care entities to recognize existing privacy related problem and devise pragmatic and actionable strategies of maintaining patient privacy in big data analytics. One paper [158] provided a policy overview of health care and data analytics, outlined the utility of health care data from a policy perspective, reviewed a variety of methods for data collection from public and private sources, mobile devices and social media, examined laws and regulations that protect data and patients’ privacy, and discussed a dynamic interplay among three aspects of today’s big data driven personal health care—policy goals to tackle both cost, population health problem and eliminate disparity in patient care while maintaining their privacy. Another study [159] proposed a Secure and Privacy Preserving Opportunistic Computing (SPOC) framework to be used in healthcare emergencies focused on collecting intensive personal health information (through mobile devices like smart phone or wireless sensors) with minimal privacy disclosure. The premise of this framework is that when a user of this system (called medical user) faces any emergency, other users in the vicinity with similar disease or symptom (if available) can come to help that user before professional help arrives. It is assumed that two persons with similar disease are skilled enough to help each other and the threshold of similarity is controlled by the user. And in physician prescribing—another paper [160] identified strategies for data mining from physicians’ prescriptions while maintaining patient privacy.

Theoretical research on personalized-health care services—treatment plans designed for someone based on the susceptibility of his/her genomic structure to a disease—also emerged from the literature review. One study [161] highlighted the potential of powerful analytical tools to open an avenue for predictive, preventive, participatory, and personalized (P4) medicine. They suggested a more nuanced understanding of the human systems to design an accurate computational model for P4 medicine. Reviewing the research paradgims of current person-centered approaches and traditions, another study [162] advocated a transdisciplinary and complex systems approach to improve the field. They synthesized the emerging aproaches and methodologies and highlighted the gaps between academic research and accessibility of evaluation, informatics, and big data from health information systems. Another paper [163] reviewed the availability of big data and the role of biomedical informatics in personalized medicine, emphasizing the ethical concerns related to personalized medicines and health equity. Personalized medicine has a potential to reduce healthcare cost, however, the researchers think it can create race, income, and educational disparity. Certain socioeconomic and demographic groups currently have less or no access to healthcare and data driven personalized medicine will exclude those groups, increasing disparities. They also highlighted the impact of EHRs and CDWs on the field of personalized medicine through acclerated research and decreased the delivery time of new technologies.

A myriad of extant theoretical points has also been identified in the literature. These topics range from exploiting big data to: study the paradigm shift in healthcare policy and management from prioritizing volume to value [164,167]; aid medical device consumers in their decision-making [166]; improve emergency departments [169]; perform command surveillance and policy analysis for Army leadership [170]; to comparing different simulation methods (i.e., systems dynamics, discrete event simulation and agent based modeling) for specific health care system problems like resource allocation, length of stay [165]; to the ethical challenges of security, management, and ownership [170]. Another researcher outlined the challenges the E.U. is facing in data mining given numerous historical, technical, legal, and political barriers [168].

## 6. Future Research and Challenges

Data mining has been applied in many fields including finance, marketing, and manufacturing [172]. Its application in healthcare is becoming increasingly popular [173]. A growing literature addresses the challenges of data mining including noisy data, heterogeneity, high dimensionality, dynamic nature, computational time. In this section, we focus on future research applications including personalized care, information loss in preprocessing, collecting healthcare data for research purposes, automation for non-experts, interdisciplinarity of study and domain expert knowledge, integration into the healthcare system, and prediction-specific to data mining application and integration in healthcare.

- Personalized care

The EMR is increasingly used to document demographic and clinician patient information [1]. EMR data can be utilized to develop personalized care plans, enhancing patient experience [162] and improving care quality.

- Loss of information in pre-processing

Pre-processing of data, including handling missing data, is the most time-consuming and costly part of data mining. The most common method used in the papers reviewed was deletion or elimination of missing data. In one study, approximately 46.5% of the data and 363 of 410 features were eliminated due to missing values [49]. In another, researchers [98] were only able to use 2064 of 4948 observations (42%) [98]. By eliminating missing value cases and outliers, we are losing a significant amount of information. Future research should focus on finding a better method of missing value estimation than elimination. Moreover, data collection techniques should be developed or modified to avoid this issue.

Similar to missing data, deletion or elimination is a common way to handle outliers [174]. However, as illustrated in one of the studies we reviewed [48], outliers can be used to gain information about rare forms of diseases. Instead of neglecting the outliers, future research should analyze them to gain insight.

- Collecting healthcare data for research purpose

Traditionally, the primary objective of data collection in healthcare is documentation of patient condition and care planning [109]. Including research objectives in the data collection process through structured fields could yield more structured data with fewer cases of error and missing values [64]. A successful example of data collection for research purpose is the Study of Health in Pomerania (SHIP) [175]. The objective of SHIP was to identify common diseases, population level risk factors, and overall health of people living in the north-east region of Germany. This study only suffered from one “mistake” for every 1000 data entries [175] which ensures a structured form of data with high reliability, less noise and fewer missing values. We can take advantage of current documentation processes (EMR or EHR) by modifying them to collect more reliable and structured data. Long-term vision and planning is required to introduce research purpose in healthcare data collection.

- Automation of data mining process for non-expert users

The end users of data mining in healthcare are doctors, nurses, and healthcare professionals with limited training in analytics. One solution for this problem is to develop an automated (i.e., without human supervision) system for the end users [134]. A cloud-based automated structure to prevent medical errors could also be developed [95]; but the task would be challenging as it involves different application areas and one algorithm will not have similar accuracy for all applications [134].

- Interdisciplinary nature of study and domain expert knowledge

Healthcare analytics is an interdisciplinary research field [134]. As a form of analytics, data mining should be used in combination with expert opinion from specific domains—healthcare and problem specific (i.e., oncologist for cancer study, cardiologist for CVD) [106]. Approximately 32% of the articles in analytics did not utilize expert opinion in any form. Future research should include members from different disciplines including healthcare.

- Integration in healthcare system

Very few articles reviewed made an effort to integrate the data mining process into the actual decision-making framework. The impact of knowledge discovery through data mining on healthcare professional’s workload and time is unclear. Future studies should consider the integration of the developed system and explore the effect on work environments.

- Prediction error and “The Black Swan” effect

In healthcare, it is better not to predict than making an erroneous prediction [46]. A little under half of the literature we identified in analytics is dedicated to prediction but, none of the articles discussed the consequence of a prediction error. High prediction accuracy for cancer or any other disease does not ensure an accurate application to decision-making.

Moreover, prediction models may be better at predicting commonplace events than rare ones [176]. Researchers should develop more sophisticated models to address the unpredictable, “The Black Swan” [176]. One study [101] addressed a similar issue in evidence based recommendations for medical prescriptions. Their concern was, how much evidence should be sufficient to make a recommendation. Many of the studies in this review do not address these salient issues. Future research should address the implementation challenges of predictive models, especially how the decision-making process should adapt in case of errors and unpredictable incidents.

## 7. Conclusions

The development of an informed decision-making framework stems from the growing concern of ensuring a high value and patient-focused health care system. Concurrently, the availability of big data has created a promising research avenue for academicians and practitioners. As highlighted in our review, the increased number of publications in recent years corroborates the importance of health care analytics to build improved health care systems world-wide. The ultimate goal is to facilitate coordinated and well-informed health care systems capable of ensuring maximum patient satisfaction.

This paper adds to the literature on healthcare and data mining (Table 1) as it is the first, to our knowledge, to take a comprehensive review approach and offer a holistic picture of health care analytics and data mining. The comprehensive and methodologically rigorous approach we took covers the application and theoretical perspective of analytics and data mining in healthcare. Our systematic approach starting with the review process and categorizing the output as analytics or theoretical provides readers with a more widespread review with reference to specific fields.

We also shed light on some promising recommendations for future areas of research including integration of domain-expert knowledge, approaches to decrease prediction error, and integration of predictive models in actual work environments. Future research should recommend ways so that the analytic decision can effectively adapt with the predictive model subject to errors and unpredictable incidents. Regardless of these insightful outcomes, we are not constrained to mention some limitations of our proposed review approach. The sole consideration of academic journals and exclusion of conference papers, which may have some good coverage in this sector is the prime limitation of this review. In addition to this, the search span was narrowed to three databases for 12 years which may have ignored some prior works in this area, albeit the increasing trend since 2005 and less number of publications before 2008 can minimize this limitation. The omission of articles published in languages other than English can also restrict the scope of this review as related papers written in other languages might be evident in the literature. Moreover, we did not conduct forward (reviewing the papers which cited the selected paper) and backward (reviewing the references in the selected paper and authors’ prior works) search as suggested by Levy and Ellis [31].

Despite these limitations, the systematic methodology followed in this review can be used in the universe of healthcare areas.

## Figures and Tables

**Table 1:** Characteristics of existing review/conceptual studies on the related topics.

Paper	Scope	Timeframe Considered	Number of Papers Reviewed
[11]	Awareness effect in type 2 diabetes	2001–2005	18
[12]	Fraud detection	N/A	N/A
[13]	Data mining techniques and guidelines for clinical medicine	N/A	N/A
[14]	Text mining, Ontologies	N/A	N/A
[15]	Challenges and future direction	N/A	N/A
[16]	Data mining algorithm, their performance in clinical medicine	1998–2008	84
[17]	Clinical medicine	N/A	N/A
[18]	Skin diseases	N/A	N/A
[19]	Clinical medicine	N/A	84
[20]	Algorithms, and guideline	N/A	N/A
[9]	Data mining process and algorithms	N/A	N/A
[21]	Algorithms for locally frequent disease in healthcare administration, clinical care and research, and training	N/A	N/A
[7]	Electronic Medical Record (EMR) and Visual analytics	N/A	N/A
[10]	Big data, Level of data usage	N/A	N/A
[22]	MapReduce architectural framework based big data analytics	2007–2014	32
[23]	Big data analytics and its opportunities	N/A	N/A
[24]	Big data analytics in image processing, signal processing, and genomics	N/A	N/A
[25]	Social media data mining to detect Adverse Drug Reaction, Natural language processing techniques (NLP)	2004–2014	39
[26]	Text mining, Adverse Drug Reaction detection	N/A	N/A
[8]	Big data analytics in critical care	N/A	N/A
[27]	Methodology of big data analytics in healthcare	N/A	N/A
**Our study**	**Application and theoretical perspective of data mining and big data analytics in whole healthcare domain**	**2005–2016**	**117**

N/A represents Not Reported.

**Table 2:** Keywords for database search.

**Phase**	**Keyword 1 (OR ^1^)**	**AND**	**Keyword 2 (OR ^1^)**
1	Healthcare, Health care		Data analysis
2	Healthcare, Health care, Cancer ^2^, Disease, Genomics		Data mining, Big data

^1^ A logical operator used between the keywords during database search. ^2^ Cancer was listed independently because other dominant associations have the word “disease” associated with them (i.e., heart disease, skin disease, mental disease etc.).

**Table 3:** Operational definition of the classes.

Class	Operational Definition *
Analytics	Knowledge discovery by analyzing, interpreting, and communicating data
3A. Types of Analytics	Data Interpretation and Communication method
Descriptive	Exploration and discovery of information in the dataset [33]
Predictive	Prediction of upcoming events based on historical data [22]
Prescriptive	Utilization of scenarios to provide decision support [22]
3B. Types of Data	Type or nature of data used in the study
Web/social media data (WS)	Data extracted from websites, blogs, social media like Facebook, Twitter, LinkedIn [23]
Sensor data (SD)	Readings from medical devices and sensors [23]
Biometric data (BM)	“Finger prints, genetics, handwriting, retinal scans, X-ray and other medical images, blood pressure, pulse and pulse-oximetry readings, and other similar types of data” [23]
Big transection data (BT)	Healthcare bill, insurance claims and transections [23]
Human generated data (HG)	Semi-structured and unstructured documents like prescription, Electronic Medical Record (EMR), notes and emails [23]
3C. Data mining techniques	Techniques applied to extract and communicate information from the dataset
Regression	Relationship estimation between variables
Association	Finding relation between variables
Classification	Mapping to predefined class based on shared characteristics
Clustering	Identification of groups and categories in data
Anomaly detection	Detection of out-of-pattern events or incidents
Data warehousing	A large storage of data to facilitate decision-making
Sequential pattern mining	Identification of statistically significant patterns in a sequence of data
3D. Application Area	Different areas in healthcare where data mining is applied for knowledge discovery and/or decision support
Clinical decision support	Analytics applied to analyze, extract and communicate information about diseases, risk for clinical use
Healthcare administration	Application of analytics to improve quality of care, reduce the cost of care and to improve overall system dynamics
Privacy and fraud detection	Privacy: Protection of patient identity in the dataset; Fraud detection: Deceptive and unauthorized activity detection
Mental health	Analytical decision support for psychiatric patients or patient with mental disorder
Public health	Analysis of problems which affect a mass population, a region, or a country
Pharmacovigilance	Post market monitoring of Adverse Drug Reaction (ADR)
3E. Theoretical study	Discusses impact, challenges, and future of data mining and big data analytics in healthcare

* Most of the definitions listed in this table are well established in literature and well know. Therefore, we did not use any specific reference. However, for some classes, specifically for types of analytics and data, varying definitions are available in the literature. We cited the sources of those definitions.

**Table 4:** Top 10 journals on application of data mining in healthcare.

Journal		Number of Articles
**1.**	Expert Systems with Applications	7
**2.**	IEEE Transection on Information Technology in Biomedicine	6
**3.**	Journal of Medical Internet Research	5
**4.**	Journal of Medical Systems	4
**5.**	Journal of the American Medical Informatics Association	4
**6.**	Health Affairs	4
**7.**	Journal of Biomedical Informatics	4
**8.**	Healthcare Informatics Research	3
**9.**	Journal of Digital Imaging	3
**10.**	PLoS ONE	3

**Table 5:** Topics and data sources of papers using clinical decision-making, organized by major disease category.

Reference	Major Disease	Topic Investigated	Data Source
[40]	Cardiovascular disease (CVD)	Risk factors associated with Coronary heart disease (CHD)	Department of Cardiology, at the Paphos General Hospital in Cyprus
[41]		Diagnosis of CHD	Invasive Cardiology Department, University Hospital of Ioannina, Greece
[42]		Classification of uncertain and high dimensional heart disease data	UCI machine learning laboratory repository
[43]		Risk prediction of Cardiovascular adverse event	U.S. Midwestern healthcare system
[44]		Cardiovascular event risk prediction	HMO Research Network Virtual Data Warehouse
[45]		Mobile based cardiovascular abnormality detection	MIT BIH ECG database
[46]		Management of infants with hypoplastic left heart syndrome	The University of Iowa Hospital and Clinics
[47]	Diabetes	Identification of pattern in temporal data of diabetic patients	Synthetic and real world data (not specified)
[48]		Exploring the examination history of Diabetic patients	National Health Center of Asti Providence, Italy
[49]		Important factors to identify type 2 diabetes control	The Ulster Hospital, UK
[50]		Comparison of classification accuracy of algorithms for diabetes	Iranian national non-communicable diseases risk factors surveillance
[51]		Type 2 diabetes risk prediction	Independence Blue Cross Insurance Company
[52]		Evaluation of HTCP algorithm in classifying type 2 diabetes patients from non-diabetic patient	Olmsted Medical Center and Mayo Clinic in Rochester, Minnesota, USA
[53]		Predicting and risk diagnosis of patients for being affected with diabetes.	1991 National Survey of Diabetes data
[54]	Cancer	Survival prediction of prostate cancer patients	The Surveillance, Epidemiology, and End Results (SEER) Program of the National Cancer Institute, USA
[38]		Classification of breast cancer patients with novel algorithm	Wisconsin Breast cancer data set, UCI machine learning laboratory repository
[42]		Classification of uncertain and high dimensional breast cancer data	UCI machine learning laboratory repository
[55]		Visualization tool for cancer	Taiwan National Health Insurance Database
[56]		Lung cancer survival prediction with the help of a predictive outcome calculator	SEER Program of the National Cancer Institute, USA
[57]	Emergency Care	Classification of chest pain in emergency department	Hospital (unspecified) emergency department EMR
[58]		Grouping of emergency patients based on treatment pattern	Melbourne’s teaching metropolitan hospital
[59]	Intensive care	Mortality rate of ICU patients	University of Kentucky Hospital
[60]		Prediction of 30 day mortality of ICU patients	MIMIC-II database
[61]	Other applications	Treatment plan in respiratory infection disease	Various health center throughout Malaysia
[62]		Pressure ulcer prediction	Cathy General Hospital (06–07), Taiwan
[63]		Pressure ulcer risk prediction	Military Nursing Outcomes Database (MilNOD), US
[64]		Association of medication, laboratory and problem	Brigham and Women’s Hospital, US
[65]		Chronic disease (asthma) attack prediction	Blue Angel 24 h Monitoring System, Tainan; Environmental Protection Administration Executive, Yuan; Central Weather Bureau Tainan, Taiwan
[66]		Personalized care, predicting future disease	No specified
[67]		Correlation between disease	Sct. Hans Hospital
[68]		Glaucoma prediction using Fundus image	Kasturba Medical college, Manipal, India
[69]		Reducing follow-up delay from image analysis	Department of Veterans Affairs health-care facilities
[70]		Disease risk prediction in imbalanced data	National Inpatient Sample (NIS) data, available at http://www.ahrq.gov by Healthcare Cost and Utilization Project (HCUP)
[71]		Survivalist prediction of kidney disease patients	University of Iowa Hospital and Clinics
[72]		Comparison surveillance techniques for health care associated infection	University of Alabama at Birmingham Hospital
[73]		Parkinson disease prediction based on big data analytics	Big data archive by Parkinson’s Progression Markers Initiative (PPMI)
[74]		Hospitalization prediction of Hemodialysis patients	Hemodialysis center in Taiwan
[75]		5 year Morbidity prediction	Northwestern Medical Faculty Foundation (NMFF)
[76]		Algorithm development for real-time disease diagnosis and prognosis	Not specified

**Table 6:** Problem analyzed and data sources in healthcare administration.

Reference	Focusing Area	Problem Analyzed	Data Source
[89]	Data warehousing and cloud computing	Developing a platform to analyze the causes of readmission	Emory Hospital, US
[90]		Development of a clinical data warehouse and analytical tools for traditional Chinese medicine	Traditional Chinese Medicine hospitals/wards
[91]		Cloud and big data analytics based cyber-physical system for patient-centric healthcare applications and services	Not specified
[92]		Repository of radiology reports	Not specified
[93]		Creation of large data repository and knowledge discovery with unsupervised learning	University of Virginia University Health System
[94]		Development of a mobile application to gather, store and provide data for rural healthcare	Not specified
[95]	Healthcare cost, quality and resource utilization	Treatment error prevention to improve quality and reduce cost	National Taiwan University Hospital
[96]		Healthcare cost prediction	US health insurance company
[97]		Healthcare resource utilization by lung cancer patients	Medicare beneficiaries for 1999, US
[98]		Length of stay prediction of Coronary Artery Disease (CAD)	Rajaei Cardiovascular Medical and Research Center, Tehran, Iran
[99]		Methodology for structured development of monitoring systems and a primary HC network resource allocation monitoring model	National Institute of Public Health; Health Care Institute, Celje; Slovenian Social Security Database, and Slovenian Medical Chamber
[100]		Assess the ability of regression tree boosting to risk-adjust health care cost predictions	Thomson Medstat’s Commercial Claims and Encounters database.
[101]		Evidence based recommendation in prescribing drugs	Dalhousie University Medical Faculty
[102]		Efficient pathology ordering system	Pathology company in Australia
[103]		Identifying people with or without insurance based on demographic and socio-economic factors	Behavioral Risk Factor Surveillance System 2004 Survey Data
[104]		Predicting care quality from patient experience	English National Health Service website
[105]	Patient management	Scheduling of patients	A south-east rural U.S. clinic
[106]		Care plan recommendation system	A community hospital in the Mid-West U.S.
[107]		Examination of risk factors to predict persistent healthcare frequent attendance	Tampere Health Centre, Finland
[108]		Forecasting number of patient visit for administrative task	Health care center in Jaen, Spain
[109]		Critical factors related to fall	1000 bed hospital in Taiwan
[110]		Verification of structured data, and codes in EMR of fall related injuries from unstructured data	Veterans Health Administration database, US
[111]	Other applications	Relation between medical school training and practice	Center for Medicare and Medicaid Service (CMS)
[112]		Analysis of physician reviews from online platform	Good Doctor Online health community
[113]		Evaluation of Key Performance Indicator (KPIs) of hospital	Greek National Health Systems for the year of 2013
[114]		Post market performance evaluation of medical devices	HCUPNet data (2002–2011)
[115]		Feasibility of measuring drug safety alert response from HC professional’s information seeking behavior	UpToDate, an online medical resource
[116]		Influencing factors of home healthcare service outcome	U.S. home and hospice care survey (2000)
[117]		Compilation of various data types for tracing, and analyzing temporal events and facilitating the use of NoSQL and cloud computing techniques	Taiwan’s National Health Insurance Research Database (NHIRD)

**Table 7:** List of papers in healthcare privacy and fraud detection.

Reference	Problem Analyzed	Data Source
[119]	Cloud based big data framework to ensure data security	Not specified
[120]	Weakness in de-identification or anonymization of health data	MedHelp and Mp and Th1 (Medicare social networking sites)
[121]	Automatic and systematic detection of fraud and abuse	Bureau of National Health Insurance (BNHI) in Taiwan.
[122]	Novel algorithm to protect data privacy	Hong Kong Red Cross Blood Transfusion Service (BTS)

**Table 8:** List of data mining application in mental health with data sources.

Reference	Problem Analyzed	Data Source
[126]	Identification and intervention of developmental delay of children	Yunlin Developmental Delay Assessment Center
[125]	Personalized treatment for anxiety disorder	Volunteer participants
[127]	Abnormal behavior detection	Through experiment with human subject
[128]	Mental health diagnosis and exploration of psychiatrist’s everyday practice	Queensland Schizophrenia Research center

**Table 9:** List of data mining application in public health with data sources.

Reference	Problem Analyzed	Data Source
[130]	Designing preventive healthcare programs	World Health Organization (WHO)
[131]	Predicting the peak of health center visit due to influenza	Military Influenza case data provided by US Armed Forces Health Surveillance Center and Environmental data from US National Climate Data Center
[132]	Contrast patient and customer loyalty, estimating Customer lifetime value, and identifying the targeted customer	Iranian Public Hospital data extracted from Hospital information system
[133]	Understanding the information seeking behavior of public and professionals on infectious disease	National electronic Library of Infection and National Resource of Infection Control, Google Trends, and relevant media coverage (LexisNexis).
[134]	Knowledge extraction for non-expert user through automation of data mining process	Brazilian health ministry
[135]	Innovative use of data mining and visualization techniques for decision-making	Slovenian national Institute of Public Health
[136]	Real-time emergency response method using big data and Internet of Things	UCI machine learning repository

**Table 10:** List of data mining application in pharmacovigilance with data sources.

Reference	Problem Analyzed	Data Source
[140]	Sentiment and network analysis based on social media data to find ADR signal	Cancer discussion forum websites
[138]	ADR signal detection from multiple data sources	Food and Drug Administration (FDA) database and publicly available electronic health record (HER) in US
[141]	ADR detection from EPR through temporal data analysis	Danish psychiatric hospital
[142]	ADR (hypersensitivity) signal detection of six anticancer agents	FDA released AERS reports (2004–2009), US
[139]	ADR caused by multiple drugs	FDA released AERS reports, US
[143]	ADR due to Statins used in Cardiovascular disease (CVD) and muscular and renal failure treatment	FDA released AERS reports, US
[144]	Creating a ranked list of Adverse Events (AEs)	EHR form European Union
[145]	Detecting ADR signals of Rosuvastatins compared to other statins users	Health Insurance Review and Assessment Service claims database (Seoul, Korea)
[146]	Unexpected and rare ADR detection technique	Medicare Benefits Scheme (MBS) and Queensland Linked Data Set (QLDS)

**Table 11:** Problem analyzed in theoretical studies.

Sector Highlight	Reference	Problem Analyzed
Disease Control, Current situation of different diseases (infection, epidemic, cancer, mental health)	[147]	Proposed an idea for dynamic clinical decision support
	[148]	Described current situation of infection control and predicted future challenges in this sector
	[149]	Described activities taken by national organization to control disease and provide better health care
	[150]	Reviewed efficient collection and aggregation of big data and proposed an intelligence based learning framework to help prevent cancer
Data quality, database framework and uncertainty quantification	[151]	Considered the management of uncertainty originating from data mining.
	[152]	Contemplated the quality of the data when collected from multimodal sources
	[150]	Provided the structure of the database of CancerLinQ that comprised of 4 key steps
	[153]	Described five major problems that need to be tackled in order to have an effective integration of big data analytics and VPH modeling in healthcare
	[152]	Discuss the issues of data quality in the context of big data health care analytics
	[154]	Discussed the necessity of proper management and confidentiality of healthcare data along with the benefit of big data analytics
Healthcare policy making	[155,156,157]	Addressed the challenges faced in implementing health care policies and considered the ethical and legal issues of performing predictive analysis on health care big data
	[150]	Focused on the US federal regulatory pathway by which CancerLinQ will have legislative authority to use the patients’ records and the approach of ASCO toward the organizing and supervising the information
Patient Privacy	[158]	Focused on ensuring patient privacy while collecting data, storing them and using them for analysis aimed to eliminate discrimination in the health care provided to patients.
	[159]	Spotted light on ensuring Privacy and security while collecting Personal Health care Information (PHI)
	[160]	Highlighted those strategies appropriate for data mining from physicians’ prescriptions while maintaining the patient’s privacy
Personalized health care	[161]	Transforming big data into computational models to provide personalized health care
	[162]	Development of informed decision-making frameworks for person centered health care
	[163]	Looked into the availability of big data and the role of biomedical informatics on the personalized medicine. Also, emphasized on the ethical concerns related to personalized medicines
Others	[164]	Finding the aspects of big data that are most relevant to Health care
	[165]	Selecting dynamic simulation modeling approach based on the availability and type of big data
	[166]	Quantifying performance in the delivery of medical services
	[167]	Identifying high risk patients to ensure better care, and explored the analytics procedure, algorithms and challenges to implement analytics
	[168]	Addressed barriers for the exploitation of health data in Europe
	[169]	Analyzed the opportunity and obstacles in applying predictive analytics based on big data in case of evaluating emergency care
	[170]	Provided an overview of the uses of the Person-Event Data Environment to perform command surveillance and policy analysis for Army leadership
	[171]	Development of big data analytics in healthcare and future challenges

## Supplementary Materials

The following are available online at http://www.mdpi.com/2227-9032/6/2/54/s1, Table S1: PRISMA checklist, Table S2: Modified checklists and comparison, Table S3: Study characteristics, Table S4: Classification of reviewed papers by analytics type, application area, data type, and data mining techniques.
