# Supplementary material for: A Systematic Review on Healthcare Analytics: Application and Theoretical Perspective of Data Mining
Source: Healthcare (Basel). 2018 May 23;6(2):54. doi: 10.3390/healthcare6020054 (PMC6023432; doi:10.3390/healthcare6020054)
Supplement: Supplementary file 1 [file healthcare-06-00054-s001.zip › healthcare-303796-supplementary/S2_Modified checklists and comparison.docx]

Supplementary Information: S2: Checklist used to assess the quality of literature

**Table S1.** Comparison of modified and original JBI Critical Appraisal Checklist for Analytical Cross Sectional Studies

| Item | Original checklist | Modified checklist |
| --- | --- | --- |
| 1 | Were the criteria for inclusion in the sample clearly defined? | Were the objective of the study and the criteria for inclusion in the sample (data selection) clearly defined? |
| 2 | Were the study subjects and the setting described in detail? | Were the sample population and variables described in details? |
| 3 | Was the exposure measured in a valid and reliable way? | Did the study detail data source (e.g. hospital, database, survey) and format (e.g. structured EMR, International Classification of Diseases code, unstructured text, survey response)? |
| 4 | Was the exposure measured in a valid and reliable way? | Was the data collected in a valid and reliable way? |
| 5 | Were confounding factors identified? | **Not applicable.**  **Replaced with:**  Was the ethical issue (patient confidentiality) considered? |
| 6 | Were strategies to deal with confounding factors stated? | **Not applicable.**  **Replaced with:**  Were findings and implications discussed in detail? |
| 7 | Were the outcomes measured in a valid and reliable way? | Were the outcomes (performance and result of data mining tools) measured in a valid and reliable way? |
| 8 | Was appropriate statistical analysis used? | Was appropriate data mining tool used? |

From: The Joanna Briggs Institute. Available online: http://joannabriggs.org/research/critical-appraisal-tools.html. (accessed on cited 07/09/2017).

Table S2. Comparison of modified and original Critical Appraisal Skills Programme (CASP) qualitative research checklist

| Item | Original checklist | Modified checklist |
| --- | --- | --- |
| 1 | Was there a clear statement of the aims of the research? | Was there a clear statement of the aims of the research? |
| 2 | Is a qualitative methodology appropriate? | Is a qualitative methodology appropriate? |
| 3 | Was the research design appropriate to address the aims of the research? | Was the research design appropriate to address the aims of the research? |
| 4 | Was the recruitment strategy appropriate to the aims of the research? | Not Applicable |
| 5 | Was the data collected in a way that addressed the research issue? | Not Applicable |
| 6 | Has the relationship between researcher and participants been adequately considered? | Not Applicable |
| 7 | Have ethical issues been taken into consideration? | Not Applicable |
| 8 | Was the data analysis sufficiently rigorous? | Not Applicable |
| 9 | Is there a clear statement of findings? | Is there a clear statement of findings? |
| 10 | How valuable is the research? | Do you consider this research valuable? |

From: Critical Approsal Skills Programme. Available online: http://docs.wixstatic.com/ugd/dded87_25658615020e427da194a325e77 73d42.pdf. (accessed on cited 07/09/2017).
