# Supplementary material for: A Systematic Review on Healthcare Analytics: Application and Theoretical Perspective of Data Mining
Source: Healthcare (Basel). 2018 May 23;6(2):54. doi: 10.3390/healthcare6020054 (PMC6023432; doi:10.3390/healthcare6020054)
Supplement: Supplementary file 1 [file healthcare-06-00054-s001.zip › healthcare-303796-supplementary/S3_Study characteristics.docx]

Supplemental Information S3: Study characteristics and quality assessment

**Table S3.** Characteristics of analytical studies

| **Title** | **Year** | **Journal** | **Keywords** | **Analytics** | **Application area** | **Problem analyzed** | **Consult domain expert?** | **Data source** | **Data type** | **Data mining method** | **Algorithm** | **Quality assessment score (out of 8)** |
| --- | --- | --- | --- | --- | --- | --- | --- | --- | --- | --- | --- | --- |
| Knowledge discovery from patients’ behavior via clustering-classification algorithms based on weighted eRFM and CLV model: An empirical study in public health care services | 2016 | Iranian Journal of Pharmaceutical Research | Hospital; Knowledge discovery; CRM; Data mining; RFM; Patient behavior | Prescriptive | Public health | Exploring contrast between patient and customer loyalty, Estimating Customer lifetime value used to segment and identifying the targeted customer | Yes | Iranian Public Hospital data extracted from Hospital information system (HIS) | Human generated data | Clustering and classification | Two step and K means algorithm for clustering, (CHAID) Decision tree algorithm for Classification. | 7 |
| Prediction of Peaks of Seasonal Influenza in Military Health-Care Data | 2016 | Biomedical Engineering and Computational Biology | Influenza; prediction; association rule mining; fuzzy logic; predictor variables | Predictive | Public health | Predicting the week of peak ILI visits per total health care visits | Yes | US Military ILI data/ Military Influenza case data provided by US Armed Forces Health Surveillance Center. US National Climate Data Center provided Environmental data | Human generated and sensor data | Classification | Running Average, Local Average of Time Series. Fuzzy Association Rule mining, Decision Tree, Random Forest and Support Vector Machine | 8 |
| Major Infection Events Over 5 Years: How Is Media Coverage Influencing Online Information Needs of Health Care Professionals and the Public? | 2013 | Journal of medical internet research | Information seeking behavior; weblogs analysis; online information needs; data mining; infectious outbreaks | Descriptive | Public health | Determining the factors that drives the search behavior of public and professionals to seek information on particular infectious disease and the correlation between this search behavior and media coverage | Yes | National electronic Library of Infection and National Resource of Infection Control  (NeLI/NRIC), (2) equivalent public online information needs (Google Trends), and (3) relevant media coverage (LexisNexis). | Website data | Association | N/A | 8 |
| A data mining system for providing analytical information on brain tumors to public health decision makers | 2013 | Computer methods and programs in bio-medicine | Data analysis; Ontology; Decision support system; Information system; Brain neoplasms; Health services; administration | Descriptive | Public health | Knowledge extraction for non-expert user through automation of data mining process | Yes | Brazilian health ministry | Human generated data | Classification, Clustering, Association | N/A | 7 |
| On Robust Methodologies for Managing Public Health Care Systems | 2014 | International journal of environmental research and public health | Diabetes; food ontologies; data warehousing; data mining; visualization; data interpretation | Description | Public health | Designing preventive health care programs | Yes | World Health Organization, WHO | Human generated data | Classification, and data warehousing | Ontology-based Warehouse Modelling | 8 |
| Data mining and visualization for decision support and modeling of public health­care resources | 2007 | Journal of biomedical informatics | Data mining; Decision support; Knowledge discovery; Knowledge management; Visualization; Applications to health-care | Description | Public health | Proposed an innovative use of data mining and visualization techniques for decision making | Yes | Slovenian national Institute of Public Health | Human generated data | Clustering | Agglomerative classification, principal component analysis, the Kolmogorov–Smirnov test, quantile range test and polar ordination | 8 |
| Real-time Medical Emergency Response System: Exploiting IoT and Big Data for Public Health | 2016 | Journal of medical systems | Big data; IoT; Healthcare; Intelligent building; Hadoop ecosystem | Descriptive | Public health | Real-time emergency response method using big data and Internet of Things | No | UCI machine learning repository | Human generated data | Classification | REPTree | 8 |
| A Comparison of Intensive Care Unit Mortality Prediction Models through the Use of Data Mining Techniques | 2011 | Healthcare informatics research | APACHE; Intensive Care Units; Neural Networks; Decision Trees; Support Vector Machines | Predictive | Clinical decision support | Mortality rate of ICU patients | Yes | University of Kentucky Hospital | Human generated data | Classification | Decision Tree, ANN, SVM | 8 |
| A Hybrid Data Mining Method for the Medical Classification of Chest Pain | 2010 | World Academy of Science, Engineering and Technology | Data mining; medical decisions; medical domain knowledge; chest pain | Predictive | Clinical decision support | Chest pain | Yes | Hospital emergency department EMR (one year, no name) | Human generated data | Association and Classification | Association rules, C5.0 for classification | 7 |
| Analysis of cancer data: a data mining approach | 2009 | Expert systems | data mining; classification; cancer; SEER; neural networks; decision trees; sensitivity analysis | Predictive | Clinical decision support | Survival prediction of prostate cancer patients | No | SEER program (2004), covers 26% of US population | Human generated data | Classification | Decision tree (CART), SVM, ANN, Logistic regression | 8 |
| Generating Treatment Plan in Medicine: A Data Mining Approach | 2009 | American Journal of Applied Sciences | SOAP format; percuro clinical information system; cross-industry standard process for data mining (CRISP-DM); international classification of disease and acute upper respiratory infection | Descriptive | Clinical decision support | Treatment plan in respiratory infection disease | No | Various health center throughout Malaysia | Human generated data | Classification | DT (C5.0) | 8 |
| Data Mining Techniques for Assisting the Diagnosis of Pressure Ulcer Development in Surgical Patients | 2011 | Journal of Medical Systems | Data mining; Pressure ulcer; Mahalanobis Taguchi System; Support vector machines; Logistic regression; Decision tree | Predictive | Clinical decision support | Pressure ulcer prediction | Yes | Cathy General Hospital (06-07), Taiwan | Human generated data | Classification | MTS, SVM, DT (C4.5), LR | 8 |
| Combining Data Mining and Discrete Event Simulation for a value-added view of a hospital emergency department | 2007 | Journal of the Operational Research Society | health service; hospital; simulation; data mining | Descriptive | Clinical decision support | Grouping of emergency patients based on treatment pattern | No | 56,906 de-identified records of all ED presentations in 2002, ED in one of Melbourne's teaching metropolitan hospital | Human generated data | Clustering | Self-organizing maps | 8 |
| A Framework for Mining Signatures from Event Sequences and Its Applications in Healthcare Data | 2013 | IEEE transactions on pattern analysis and machine intelligence | Temporal signature mining; sparse coding; dictionary learning; nonnegative matrix factorization; stochastic gradient descent; beta-divergence | Descriptive | Clinical decision support | Identification of pattern in temporal data | No | Synthetic data and real world data; EMR (source not mentioned) | Human generated data | Sequential pattern function | Novel algorithm | 7 |
| Assessment of the Risk Factors of Coronary Heart Events Based on Data Mining With Decision Trees | 2010 | IEEE transactions on information technology in biomedicine | Coronary heart disease (CHD); data mining; decision trees; risk factors | Descriptive | Clinical decision support | Risk factors associated with Coronary heart disease (CHD) | No | Data from 1500 consecutive CHD subjects were collected 2003–2006 and 2009 (300 subjects each year)  at the Department of Cardiology; at the Paphos General Hospital in Cyprus | Human generated data | Classification | DT (C4.5) | 8 |
| A new hybrid approach for mining breast cancer pattern using discrete particle swarm optimization and statistical method | 2009 | Expert Systems with Applications | Breast cancer; Classification rules; Statistical method; Discrete particle swarm optimization | Predictive | Clinical decision support | Classification of breast cancer patients with novel algorithm | No | Wisconsin Breast cancer data set (benchmark data) available at UCI machine learning repository | Human generated data | Classification | Novel algorithm (PSO based hybrid algorithm) | 8 |
| Adapting machine learning techniques to censored time-to-event health record data: A general-purpose approach using inverse probability of censoring weighting | 2016 | Journal of Biomedical Informatics | Censored data; Electronic health data; Inverse probability; weighting; Machine learning; Risk prediction; Survival analysis | Predictive | Clinical decision support | Risk prediction of Cardiovascular adverse event | Yes | U.S. Midwestern healthcare system | Human generated data | Classification | Bayesian Network; K-NN; Classification Tree; Logistic regression model and Generalized adaptive model | 8 |
| An automated technique for identifying associations between medications; laboratory results and problems | 2010 | Journal of Biomedical Informatics | Data mining; Association rule mining; Clinical decision support | Descriptive | Clinical decision support | Association of medication; laboratory and problem | Yes | Brigham and Women’s Hospital; US | Human generated data | Association | Frequent item list and apriory | 8 |
| A novel data mining mechanism considering bio-signal and environmental data with applications on asthma monitoring | 2011 | Computer methods and programs in bio-medicine | Data mining; Bio-signal analysis; Environmental factors; Patient monitoring; Asthma attacks | Predictive | Clinical decision support | Chronic disease attack prediction (asthma attack) | Yes | Blue Angel 24 h Monitoring System of Tainan Allergic Asthma Infants Health Association; Environmental Protection Administration Executive Yuan; Central Weather Bureau Tinan; Taiwan | Human generated data; website data | Classification | Pattern based decision tree and pattern based association rule | 8 |
| Automated Diagnosis of Coronary Artery Disease Based on Data Mining and Fuzzy Modeling | 2008 | IEEE transactions on information technology in biomedicine | Coronary artery disease (CAD); data mining; decision trees; fuzzy modeling; optimization | Predictive | Clinical decision support | Coronary heart disease | Yes | Invasive Cardiology Department of the University Hospital of Ioannina; Greece | Human generated data | Classification | DT (C 4.5); Fuzzy classification model | 8 |
| Bringing Big Data to Personalized Healthcare: A Patient-Centered Framework | 2013 | Journal of general internal medicine | personalized healthcare; data mining; patient-centered outcomes | Predictive | Clinical decision support | Personalized care; predicting future disease | No | N/A | Human generated data | Clustering | N/A | 6 |
| Using Electronic Patient Records to Discover Disease Correlations and Stratify Patient Cohorts | 2011 | PLOS Computational Biology | N/A | Descriptive | Clinical decision support | Correlation between diseases | Yes | Sct. Hans Hospital | Human generated data | Clustering | N/A | 7 |
| Analysis of diabetic patients through their examination history | 2013 | Expert Systems with Applications | data mining; cluster analysis; patient examination history; diabetes | Descriptive | Clinical decision support | Exploring the examination history of Diabetic patients | Yes | National Health Center of Asti Province in Italy | Human generated data | Clustering | ABSCAN | 8 |
| Classification of healthcare data using genetic fuzzy logic system and wavelets | 2015 | Expert Systems with Applications | Fuzzy standard additive model; Genetic algorithm; Wavelet transformation; Healthcare data classification; Breast cancer; Heart disease | Predictive | Clinical decision support | Developed an algorithm and applied it to two benchmark data set (heart and cancer data set) | No | UCI machine learning respiratory | Human generated data | Classification | Novel algorithm (Combined Fuzzy and GA) | 8 |
| Feature selection and classification model construction on type 2 diabetic patients’ data | 2007 | Artificial Intelligence in Medicine | Type 2 diabetes; Blood glucose; Data mining; Classification; Feature selection | Descriptive | Clinical decision support | Important factors (feature selection) to identify type 2 diabetes control | Yes | The Ulster Hospital; UK | Human generated data | Classification | Naïve Bayes; IB1 and C4.5 for classification. FSSMC for feature selection | 8 |
| Data mining technique for automated diagnosis of glaucoma using higher order spectra and wavelet energy features | 2012 | Knowledge-based systems | Glaucoma; Retina imaging; Computer aided diagnosis; Higher order spectra; Wavelet; Support vector machine | Predictive | Clinical decision support | Glaucoma prediction using Fundus image | Yes | Kasturba Medical college; Manipal; India (60 fundus images; 30 normal 30 glaucoma) | Biometric data | Classification | SVM (feature selection was done by DWT and HOS) | 8 |
| Exploring factors associated with pressure ulcers: A data mining approach | 2015 | International Journal of Nursing Studies | Data mining; Predictive modeling; Pressure ulcers; Braden scale | Predictive | Clinical decision support | Pressure ulcer risk perdition | Yes | Military Nursing Outcomes Database (MilNOD); US | Human generated data | Classification | DT; Random forest; MARS; LR | 8 |
| Real-Data Comparison of Data Mining Methods in Prediction of Diabetes in Iran | 2013 | Healthcare informatics research | Diabetes; Cluster Sampling; Data Mining; Support Vector Machine; Logistic Regression | Predictive | Clinical decision support | Compared classification accuracy of algorithms for diabetes | Yes | Iranian national non-communicable diseases risk factors surveillance (2005-09) | Human generated data | Classification | SVM; ANN; Fuzzy C-mean; RF; LR; Fishers LDA) | 8 |
| Predicting disease risks from highly imbalanced data using random forest | 2011 | BMC Medical Informatics and Decision Making | N/A | Predictive | Clinical decision support | Disease risk prediction in imbalanced data | Yes | National Inpatient Sample (NIS) data; which is publicly available through Healthcare Cost and Utilization Project (HCUP) | Human generated data | Classification | RF; SVM; Bagging and Boosting | 8 |
| Predicting survival time for kidney dialysis patients: a data mining approach | 2005 | Computers in Biology and Medicine | Hemodialysis; Survival; Data mining; Data preprocessing; Data transformations; Decision making; Medical relevance; Dialysis protocol | Predictive | Clinical decision support | Survival prediction of kidney disease patients | Yes | University of Iowa Hospital and Clinics | Human generated and sensor data | Classification | DT; Rough set | 8 |
| Data mining for censored time-to-event data: a Bayesian network model for predicting cardiovascular risk from electronic health record data | 2014 | Data Mining and Knowledge Discovery | N/A | Predictive | Clinical decision support | CV event risk prediction | Yes | HMO Research Network Virtual Data Warehouse (HMORN VDW) from a healthcare system from the Midwestern U.S | Human generated data and Big transection data | Classification | Bayesian network | 8 |
| Diagnosis of Cardiovascular Abnormalities From Compressed ECG: A Data Mining-Based Approach | 2011 | IEEE transactions on information technology in biomedicine | Cardiac abnormality detection; faster cardiovascular diagnosis; m-health; medical data mining; mobile telecardiology | Descriptive | Clinical decision support | Mobile based cardiovascular abnormality detection | No | MIT BIH ECG database | Sensor generated data | Clustering | EM; Correlation based feature selection | 8 |
| Performance Evaluation of Weighted Associative Classifier in Health Care Data Mining and Building Fuzzy Weighted Associative Classifier | 2011 | Advances in parallel; distributed computing | Associative Classifiers; Weighted Associative Classifiers; Association Rule Mining; Classifiers; Prediction accuracy | Predictive | Clinical decision support | Building Fuzzy Weighted Associative Classifier | No | UCI Machine learning repository | Human generated data | Classification | Weighted Associative Classifiers/ Fuzzy Weighted Associative Classifiers | 8 |
| A comparison of 3 metrics to identify health care-associated infections | 2012 | American Journal of Infection Control | Device-associated infections; Surveillance; Public reporting | Descriptive and Prediction | Clinical decision support | Comparison of traditional surveillance; MedMined Data Mining Surveillance and Administrative coding to measure healthcare associated infection | Yes | Hospital Microbiology Laboratory | Human generated data | Classification | N/A | 6 |
| Hypoplastic left heart syndrome: knowledge discovery with a data mining approach | 2006 | Computers in Biology and Medicine | Hypoplastic left heart syndrome; Data mining; Medical knowledge discovery; Classification accuracy; Classification quality; Medical decision making | Predictive | Clinical decision support | Management of infants with hypoplastic left heart syndrome | Yes | The University of Iowa Hospital and Clinics | Sensor data and Human generated | Classification | Rough set | 8 |
| Cancer-disease associations: A visualization and animation through medical big data | 2016 | Computer methods and programs in biomedicine | Visual analytics; Disease visualization; Big data visualization; Cancer disease visualization; Cancer comorbidities visualization | Descriptive | Clinical decision support | To build an animated medical data visualization tool which provides a dynamic; time-lapse; animated view of cancer-disease associations across different age groups and gender | Yes | Taiwan National Health Insurance Database | Human generated data | Association | N/A | 7 |
| Computerized Triggers of Big Data to Detect Delays in Follow-up of Chest Imaging Results | 2016 | Chest | Electronic health records; health information technology; lung cancer; medical informatics; primary care; radiology; triggers | Predictive | Clinical decision support | Design triggers to identify patients experiencing delays in diagnostic evaluation of chest imaging results suspicious for malignancy | Yes | Department of Veterans Affairs health-care facilities | Human generated data | Classification | Trigger (decision tree) | 8 |
| Personalized Mortality Prediction Driven by Electronic Medical Data and a Patient Similarity Metric | 2015 | PLOSE ONE | N/A | Predictive | Clinical decision support | Predict 30-day mortality rate | No | MIMIC-II database | Human generated data | Classification | Logistic regression; Decision tree | 8 |
| Population-Level Prediction of Type 2 Diabetes From Claims Data and Analysis of Risk Factors | 2015 | Big data | Big data analytics; data mining; machine learning; predictive analytics; risk assessment; disease prediction; longitudinal study | Predictive | Clinical decision support | New approach to predict type 2 diabetes | No | Independence Blue Cross Insurance company | Human generated data | Classification | Sparse logistic regression | 8 |
| Predictive Big Data Analytics: A Study of Parkinson’s Disease Using Large; Complex; Heterogeneous; Incongruent; Multi-Source and Incomplete Observations | 2016 | PLOSE ONE | N/A | Predictive | Clinical decision support | Propose, implement, test, and validate complementary model-based and model-free approach for Parkinson's Disease classification and prediction | No | PPMI | Human generated data | Classification | AdaBoost; support vector machine; Naïve Bayes; Decision Tree; KNN; and K-Means | 8 |
| Using data mining techniques to predict hospitalization of hemodialysis patients | 2011 | Decision support systems | Hemodialysis; Temporal abstract; Data mining; Healthcare quality | Predictive | Clinical decision support | Predicting hospitalization of Hemodialysis patients to determine the service quality of the health care; precisely hemodialysis department | No | Hemodialysis center in Taiwan | Human generated data | Sequential pattern mining | Temporal abstraction integrated with association rule mining and Decision Tree and Msapriori algorithm | 8 |
| Real-Time Clinical Decision Support System with Data Stream Mining | 2012 | Journal of Biomedicine and Biotechnology | N/A | Predictive | Clinical decision support | Combining software technology and medical functions for the development of software application that can be used for real time chronic disease prognosis and diagnosis. | No | N/A | Human generated data | Classification | Improved Very Fast Decision Tree (VFDT). | 6 |
| Lung cancer survival prediction using ensemble data mining on SEER data | 2012 | Scientific programming | Ensemble data mining; lung cancer; predictive modeling; outcome calculator | Predictive | Clinical decision support | Predicting lung cancer with the help of developing a predictive outcome calculator. | No | The Surveillance; Epidemiology; and End Results (SEER) Program of the National Cancer Institute; USA | Human generated data | Classification | J48 decision tree; Random forest; LogitBoost; Random subspace; Alternating decision tree | 8 |
| Impact of data fragmentation across healthcare centers on the accuracy of a high-throughput clinical phenotyping algorithm for specifying subjects with type 2 diabetes mellitus | 2012 | Research and Applications | N/A | Predictive | Clinical decision support | Evaluating the accuracy of the high throughput clinical phenotyping (HTCP) algorithm in analyzing the data fragmentation across health care centers | Yes | Clinical decision making Used provider-linked electronic medical record data from the two healthcare centers that provide >95% of all care to County residents (i.e. Olmsted Medical Center and Mayo Clinic in Rochester; Minnesota; USA). | Human generated data | Classification | High throughput clinical phenotyping (HTCP) algorithm | 8 |
| Intelligible Support Vector Machines for Diagnosis of Diabetes Mellitus | 2010 | IEEE Transactions on Information Technology in Biomedicine | Data mining; Diabetes; Machine learning; Medical diagnosis. | Predictive | Clinical decision support | Predicting and Diagnosing the risk of patients for being affected by diabetes. | Yes | 1991 National Survey of Diabetes data | Human generated data | Classification followed by clustering | K-Means clustering algorithm and SVM rules Extraction | 8 |
| Development of a 5 year life expectancy index in older adults using predictive mining of electronic health record data | 2013 | Journal of the American Medical Informatics Association | N/A | Predictive | Clinical decision support | 5 year Morbidity prediction | Yes | Northwestern Medical Faculty Foundation (NMFF) during 2003 | Human generated data | Classification | Rotation forest ensembling with alternating decision tree | 8 |
| A data mining approach in home healthcare: outcomes and service use | 2006 | BMC Health Services Research | Systems; HL7 | Descriptive | Administrative | Influencing factors of home healthcare service outcome | Yes | 2000 National home and hospice care survey (NHHCS) | Human generated data | Classification | Classification and Regression Tree (CART) | 8 |
| Analysis of healthcare coverage: A data mining approach | 2009 | Expert Systems with Applications | Healthcare coverage; Data mining; Prediction; Classification; Neural networks; Decision trees | Predictive | Administrative | Identify people with or without insurance based on different factors | No | Behavioral Risk Factor Surveillance System 2004 Survey Data (Healthy People 2010; 2000) | Human generated data | Classification | ANN (multi-layered perceptron); DT (CART) | 8 |
| A Data-Mining Framework for Transnational Healthcare System | 2012 | Journal of Medical Systems | Transnational; Order sets; Clinical pathway; Clinical guideline; Service-oriented; SOA; Data mining | Predictive | Administrative | Treatment error prevention to improve quality and reduce cost | Yes | National Taiwan University Hospital | Human generated data | Clustering | Sequential clustering | 7 |
| A hybrid data mining/simulation approach for modelling outpatient no-shows in clinic scheduling | 2009 | Journal of the Operational Research Society | Outpatient scheduling; healthcare; data mining; association rules; simulation | Predictive | Administrative | Scheduling of patients | No | A south-east rural American clinic | Human generated and Sensor data | Classification | Association rule mining | 8 |
| Algorithmic Prediction of Health-Care Costs | 2008 | Operations research | Health care; cost predictions; prediction algorithms; claims data | Prediction | Administrative | Healthcare cost prediction | Yes | US health insurance company | Big transection data | Classification and Clustering | Classification Tree; Unknown for Clustering | 8 |
| Application of data mining to the identification of critical factors in patient falls using a web-based reporting system | 2011 | International journal of medical informatics | Nursing; Incidents; Falls; Information system; Data mining | Descriptive | Administration | Critical factors related to fall | Yes | 1000 bed hospital in Taiwan | Human generated data | Classification | ANN; Stepwise logistic regression | 8 |
| The Analytic Information Warehouse (AIW): A platform for analytics using electronic health record data | 2013 | Journal of Biomedical Informatics | Healthcare analytics; Clinical data warehousing; Temporal abstraction; Quality improvement; Comparative effectiveness | Prescriptive | Administration | Developing a platform for analytics; cause of readmission | Yes | Emory hospital; US | Human generated data | Data warehouse system | N/A | 8 |
| Development of traditional Chinese medicine clinical data warehouse for medical knowledge discovery and decision support | 2010 | Artificial Intelligence in Medicine | Clinical data warehouse; Traditional Chinese medicine; Clinical data mining; Clinical decision support | Prescriptive | Administration | Developed a clinical data warehouse for traditional Chinese medicine; provides analytical tools | Yes | Traditional Chinese Medicine hospitals/wards | Human generated data | Data warehouse system | N/A | 8 |
| Mining lung cancer patient data to assess healthcare resource utilization | 2008 | Expert Systems with Applications | Data mining; Propensity score; Medicare claims data; Lung cancer; Healthcare utilization | Descriptive | Administrative | Healthcare resource utilization by lung cancer patients | Yes | Medicare beneficiaries for 1999; US | Big transection data | Classification | DT; ANN | 8 |
| Health-CPS: Healthcare Cyber-Physical System Assisted by Cloud and Big Data | 2015 | IEEE Systems journal | Body area networks (BANs); big data; cloud computing; cyber-physical systems (CPS); healthcare | Prescriptive | Administrative | Cloud and big data analytics based cyber-physical system for patient-centric healthcare applications and services | No | N/A | Human generated data | Data warehousing | N/A | 7 |
| Does Medical School Training Relate to Practice? Evidence from Big Data | 2015 | Big Data | Big data analytics; data acquisition and cleaning; data mining | Descriptive | Administrative | Relation between medical school training and practice | No | Center for Medicare and Medicaid Service (CMS) | Human generated data | Clustering | K-means | 8 |
| Healthcare information systems: data mining methods in the creation of a clinical recommender system | 2011 | Enterprise Information Systems | Nursing care plan; recommender system; data mining; correlation; information value; medical informatics; healthcare integrated information systems; healthcare enterprise-wide systems | Prescriptive | Administration | Care plan recommendation system | No | A Community hospital in Mid-West | Human generated data | Association | Prefix tree search | 7 |
| Use of Data Mining Techniques to Determine and Predict Length of Stay of Cardiac Patients | 2013 | Healthcare Informatics Research | Length of Stay; Data Mining; Coronary Artery Disease; Patients; Extract | Predictive | Administration | LOS prediction of CAD | Yes | Academic and Educational Hospital of Rajaei Cardiovascular Medical & Research Center in Tehran; Iran | Human Generated data | Classification | SVM; C5.0; ANN | 8 |
| Identifying fall-related injuries: Text mining the electronic medical record | 2009 | Information Technology and Management | Healthcare informatics; Electronic medical records; Text mining; Cluster analysis; Latent semantic indexing; Veterans administration | Predictive | Administration | Verification of structured data (codes entered in EMR) from unstructured data (clinical note) by text mining in fall related injuries | Yes | Veterans Health Administration (VHA) database; US | Human generated data | Clustering and Classification | K-means (clustering); LR (classification ) | 8 |
| Development of a Google-Based Search Engine for Data Mining Radiology Reports | 2009 | Journal of Digital Imaging | Google; data mining; reports; HIPAA; search engine | Prescriptive | Administration | Repository of radiology reports | Yes | N/A | Human generated data | Data warehousing | N/A | 7 |
| Data mining and clinical data repositories: Insights from a 667;000 patient data set | 2006 | Computers in Biology and Medicine | Clinical data repository; Complex data sets; Large patient cohort; FANO; HealthMiner ; Search tools | Descriptive | Administrative | Creation of large data repository and knowledge discovery with unsupervised learning | Yes | University of Virginia University health system | Human generated data | Association; classification | N/A | 7 |
| Geospatial technology in disease mapping; e-surveillance and health care for rural population in south India | 2014 | The International Archives of the Photogrammetry | Health care; GIS; Data mining; Visualization; Risk analysis; ICT | Description | Administrative | Describe a mobile application that help to gather; store and provide data for rural healthcare | Yes | N/A | Human generated data | Classification | Decision tree | 6 |
| Primary healthcare network monitoring: a hierarchical resource allocation modeling approach | 2010 | International journal of health planning and management | Primary health-care network; resource allocation; health-care disparities; data analysis; data mining | Description | Administration | Proposed a methodology for structured development of monitoring systems and a PHCN resource allocation monitoring model | Yes | National Institute of Public Health; Health Care Institute Celje; Slovenian Social Security Database; and Slovenian Medical Chamber | Human generated data | Association | Association rules | 7 |
| Risk factors for persistent frequent use of the primary health care services among frequent attenders: A Bayesian approach | 2010 | Scandinavian journal of primary health care | Data mining; decision-making; family practice; follow-up studies; frequent attender; health services research/utilization; prognosis/methods | Descriptive and Predictive | Administration | Examine risk factors that predict persistent healthcare frequent attendance | Yes | Tampere Health Centre | Human generated data | Classification | Naive Bayes | 8 |
| Regression Tree Boosting to Adjust Health Care Cost Predictions for Diagnostic Mix | 2008 | Health services research | Risk adjustment; case mix; health care cost; boosting; data mining | Descriptive and Predictive | Administration | Assess the ability of regression tree boosting to risk-adjust health care cost predictions | Yes | Thomson Medstat’s Commercial Claims and Encounters database. | Human generated data | Classification | Boosted regression tree | 8 |
| “Big data” in health care Assessment of the performance of Greek NHS hospitals using key performance and clinical workload indicators | 2016 | Archives of Hellenic Medicine | Health big data; Clinical workload indicators; Hospital performance; Key performance indicators | Descriptive | Administration | How common KPIs change when hospitals critical workloads are changed. | Yes | Recorded data form 129 Greek National Health Systems for the year of 2013. Data was recorded by the ESY.net web application. DRG data were collected directly from the hospitals website. | Website data | Clustering | N/A | 6 |
| An in silico framework for integrating epidemiologic and genetic evidence with health care applications: ventilation-related pneumothorax as a case illustration | 2016 | Journal of the American Medical Informatics Association | In silico evidence integration using big data approaches; Translational Epidemiology; Repurposing and re-utilization of pre-existing genetic data; Medical device safety biomarkers | Descriptive and Predictive | Administration | How to integrate epidemiologic and genetic evidence in the research of evaluating post market medical device performance. | Yes | Healthcare Research and Quality (AHRQ)/Healthcare Cost and Utilization Project (HCUPNet) data (2002–2011) | Website data | Classification, Regression | N/A | 7 |
| Analyzing Information Seeking and Drug Safety Alert Response by Health Care Professionals as New Methods for Surveillance | 2015 | Journal of Medical Internet Research | Internet log analysis; data mining; physicians; information-seeking behavior; drug safety surveillance | Descriptive | Administration | Analyzing health care professional's information seeking behavior and assesse the feasibility of measuring drug safety alert response by using the website logs data. | Yes | Logs from UpToDate website | Website data | Association | Unitex Corpus processor; Time Stamps; | 8 |
| An Improvement in the Appointment Scheduling in Primary Health Care Centers Using Data Mining | 2014 | Journal of Medical Systems | Appointment scheduling; Primary health care; Data mining | Predictive | Administration | Predicting how many patients will visit health center and scheduling them; keeping in mind that time required for administrative task is far less than the clinical task | Yes | Health care center in Jaen; Spain | Human generated and website data | Regression | Generalized Linear Models (GLM); Support Vector Machines (SVM) with linear and Gaussian Kernel Algorithms | 8 |
| Finding best evidence for evidence based best practice recommendations in health care: the initial decision support system design | 2011 | Knowledge and information systems | Evidence-based best practices; Decision support; Naive Bayes; Segment-based hidden Markov models (HMMs); Multiply sectioned Bayes nets; Association rules; Reduct; Information extraction; Data mining | Descriptive | Administration | How to find best evidence to develop an evidence based decision making framework and consequently prescribing best drugs to the patients in a cost effective way strengthened by improved patient quality and optimized drug related outcomes. | Yes | Dalhousie University Medical Faculty. | Human generated data | Classification; Association | Naïve Bayesian Networks; Hidden Markov decision Model | 8 |
| The Voice of Chinese Health Consumers: A Text Mining Approach to Web-Based Physician Reviews | 2016 | Journal of medical internet research | Online doctor review; physician ratings; text mining; China health consumers | Descriptive | Administration | Using an automated text-mining approach to analyze a large amount of unstructured textual data of Web-based physician reviews in China | No | Good Doctor Online | Website data | Clustering | Latent Dirichlet Allocation | 7 |
| Temporal Event Tracing on Big Healthcare Data Analytics | 2016 | Big Data Applications and Use Cases | Big medical data; NoSQL; temporal event analysis; shard; data mining; medical record | Descriptive | Administration | How to compile various data types for tracing and analyzing temporal events in addition to facilitate the use of NoSQL and cloud computing techniques to rapidly acquire; store; process and analyze the health big data. | Yes | Taiwan’s National Health Insurance Research Database (NHIRD) | Website data | Association | Query map reducing functions and sharding-key techniques | 8 |
| Use of Sentiment Analysis for Capturing Patient Experience From Free-Text Comments Posted Online | 2013 | Journal of medical internet research | Internet; patient experience; quality; machine learning | Predictive | Administration | How to use patient's unstructured free text information available in internet; social media; blogs and physicians rating website to predict the quality of health care. | Yes | English National Health Service website | Website data | Classification | Naïve Bayes multinomial (NBM); Decision Trees; Bagging; SVM or Support Vector Machine | 8 |
| Combining Data Mining and Case-based Reasoning for Intelligent Decision Support for Pathology Ordering by General Practitioners | 2009 | European Journal of Operational Research | Decision Support; Data Mining; Case Based Reasoning; Data Clustering; Kohonen’s Self Organizing Maps; Health Care Systems | Descriptive | Administration | Efficient pathology ordering system | Yes | XYZ pathology company in Australia | Human generated data | Clustering | Kohonen's self-organizing maps | 8 |
| A framework for secure healthcare systems based on big data analytics in mobile cloud computing environments | 2014 | International Journal of Ambient Systems and Applications | Electronic Health Records; Big Data; Mobile Cloud Computing; Information Security; Healthcare | Prescriptive | Data privacy | Cloud based big data framework | Yes | N/A | Human generated data | Data warehousing | N/A | 7 |
| New threats to health data privacy | 2011 | BMC Bioinformatics | N/A | Descriptive | Data privacy | New threat to healthcare data privacy | No | MedHelp and Mp & Th1 (Medicare social networking sites) | Website data | Association | N/A | 7 |
| A process-mining framework for the detection of healthcare fraud and abuse | 2006 | Expert Systems with Applications | Healthcare fraud; Healthcare abuse; Clinical pathways; Classification model; Data mining | Descriptive | Privacy and Fraud detection | Automatic and systematic detection of fraud and abuse | Yes | Bureau of National Health Insurance (BNHI) in Taiwan. | Big transection data | Classification | Classification Based on Associations algorithm (CBA) | 8 |
| Centralized and Distributed Anonymization for High-Dimensional Healthcare Data | 2010 | ACM Transactions on Knowledge Discovery from Data | Privacy; anonymity; classification; healthcare | Descriptive | Privacy and Fraud detection | New algorithm to protect data privacy | Yes | Hong Kong Red Cross Blood Transfusion Service (BTS) | Human generated data | Classification | Novel Algorithm | 7 |
| A study of applying data mining to early intervention for developmentally-delayed children | 2007 | Expert system with applications | Data mining; Developmental delay; Decision tree; Association rule | Descriptive | Mental health | Developmental delay of children | No | Yunlin Developmental Delay Assessment Center from 2003 to 2005 | Human generated data | Classification and Association | Decision tree (C 5.0); Association rule | 8 |
| A Contextual Data Mining Approach Toward Assisting the Treatment of Anxiety Disorders | 2010 | IEEE transactions on information technology in biomedicine | Context awareness; machine learning; mental health; user modeling | Prescriptive | Mental health | Personalized treatment for anxiety disorder | Yes | Volunteer participants | Human generated data | Association and Classification | Apriori association and Bayesian network classification | 8 |
| An automatic data mining method to detect abnormal human behavior using physical activity measurements | 2014 | Pervasive and Mobile Computing | Automatic method; Abnormal human behavior; Data mining method; Real time analysis; Physical activity measurement | Prescriptive | Mental health | Abnormal behavior detection | No | Through experiment with human subject | Sensor data | Anomaly detection | N/A | 7 |
| E -ray: Data mining and mental health | 2007 | Applied soft computing | Machine learning; Text classification; Mental health; Psychiatry; Diagnosis | Predictive (Exp 1); Descriptive (Exp 2) | Mental health | Classification of free speech text; Exploration of psychiatric diagnosis | No | Queensland Schizophrenia Research center | Human generated data | Classification and Clustering | SVM; DT for classification and Hierarchical clustering | 8 |
| Network-Based Modeling and Intelligent Data Mining of Social Media for Improving Care | 2015 | IEEE journal of biomedical and health informatics | N/A | Descriptive | Pharmacovigilance | Sentiment and network analysis based on social media data to find ADR signal | No | Cancer discussion forums | Social media data | Clustering | Self-Organizing Maps | 7 |
| Combing signals from spontaneous reports and electronic health records for detection of adverse drug reactions | 2013 | Journal of the American Medical Informatics Association | N/A | Descriptive | Pharmacovigilance | ADR signal detection from multiple data sources | Yes | Food and Drug Administration and publicly available HER | Human generated data | Association | MGPS | 8 |
| Dose-Specific Adverse Drug Reaction Identification in Electronic Patient Records: Temporal Data Mining in an Inpatient Psychiatric Population | 2014 | Drug Safety | N/A | Descriptive | Pharmacovigilance | ADR detection from EPR through temporal data analysis | Yes | tertiary Danish psychiatric hospital | Human generated data | Association | N/A | 7 |
| Hypersensitivity reactions to anticancer agents: Data mining of the public version of the FDA adverse event reporting system; AERS | 2011 | Journal of Experimental & Clinical Cancer Research | N/A | Descriptive | Pharmacovigilance | ADR (hypersensitivity) signal detection of 6 anticancer agents | Yes | Food and Drug Administration Released AERS (2004-2009); US | Human Generated data | Association | Information component | 8 |
| Mining multi-item drug adverse effect associations in spontaneous reporting systems | 2010 | BMC Bioinformatics | N/A | Descriptive | Pharmacovigilance | ADR caused by multiple drugs | Yes | FDA's AERS reports; US | Human generated data | Association | Modified Apriori algorithm | 7 |
| Statin-Associated Muscular and Renal Adverse Events: Data Mining of the Public Version of the FDA Adverse Event Reporting System | 2011 | PLOS ONE | N/A | Descriptive | Pharmacovigilance | Association of Statins used in Cardiovascular disease (CVD) and muscular & renal failure | No | FDA's AERS reports; US | Human generated data | Association | Proportional reporting ratio (PRR); reporting odds ratio (ROR); information component (IC); and empirical Bayes geometric mean (EBGM) | 8 |
| Data mining on electronic health record databases for signal detection in pharmacovigilance: which events to monitor? | 2009 | Pharmacoepidemiology and drug safety | Adverse event; data mining; drug safety; database; signal detection | Descriptive | Pharmacovigilance | Creating a ranked list of AEs | Yes | Electronic HR form European Union | Human generated data | Association | N/A | 7 |
| Signal detection of rosuvastatin compared to other statins: Data-mining study using national health insurance claims database | 2010 | Pharmacoepidemiology and Drug Safety | Pharmacovigilance; data-mining; health insurance claims database; relative risk; rosuvastatin | Descriptive | Pharmacovigilance | Detecting adverse drug reaction signals of rosuvastatins compared to other statins users | No | Health Insurance Review & Assessment Service (HIRA) claims database (Seoul; Korea) | Big transection data | Association | Relative Risk (RR) Based Datamining Approach | 7 |
| Mining Unexpected Temporal Associations: Applications in Detecting Adverse Drug Reactions (done) | 2008 | IEEE transactions on information technology in biomedicine | Adverse drug reaction (ADR); data mining; healthcare administrative databases; pharmacovigilance; unanticipated episode; unexpected temporal association. | Descriptive | Pharmacovigilance | How to describe unexpected episodes that lead to infrequent and unexpected patters in data. | Yes | Medicare Benefits Scheme (MBS) and Queensland Linked Data Set (QLDS) | Human generated data | Association | MUTARC | 8 |

**Table 2: Characteristics of theoretical studies**

| **Title** | **Year** | **Journal** | **Keywords** | **Sector** | **Problem analyzed** | **Consult domain expert?** | **Quality assessment score (out of 5)** |
| --- | --- | --- | --- | --- | --- | --- | --- |
| Dynamic Clinical Data Mining: Search Engine-Based Decision Support | 2014 | JMIR medical informatics | Decision support; clinical informatics; big data | Disease control | Proposed an idea for Dynamic Clinical decision support | Yes | 5 |
| Uncovering and Improving Upon the Inherent Deficiencies of Radiology Reporting through Data Mining | 2010 | Journal of Digital Imaging | Uncertainty, reporting, data mining | Data quality and uncertainty | Use of data mining in uncertainty qualification of radiology report | Yes | 5 |
| Infection control and quality health care in the new millenium | 2005 | American journal of infection control | N/A | Disease (infection) control | Discuss about infection control | Yes | 5 |
| Transforming big data into computational models for personalized medicine and health care | 2016 | Dialogues in clinical neuroscience | Big data; challenges; computational method; health care system; personalized medicine | Personalized healthcare | How big data can be transformed into computational model to provide personalized healthcare | Yes | 5 |
| Making value a priority: how this paradigm shift is changing the landscape in health care | 2016 | Annals of the New York academy of sciences | value in health care; revolution; paradigm shift | Overall healthcare situation | Highlight the recent changes taken place on healthcare. | Yes | 4 |
| A national action plan for sharable and comparable nursing data to support practice and translational research for transforming health care | 2015 | Journal of the American Medical Informatics Association | nursing informatics; terminology; electro health records; consensus development conference; national health policy | Nursing data integration with Electronic Health Records | Focused on the development of national action plan by integrating standardized nursing data with Electronic Health Records | Yes | 5 |
| Selecting a Dynamic Simulation Modeling Method for Health Care Delivery Research Part 2: Report of the ISPOR Dynamic Simulation Modeling Emerging Good Practices Task Force | 2015 | Value in Health | Decision making; dynamic simulation modeling; healthcare delivery methods | Health care delivery systems problems that can be addressed by Dynamic Simulation modeling | Which Dynamic simulation modeling approach should be selected for particular health care problem on hand | Yes | 5 |
| Quality of Big Data in health care | 2015 | International Journal of Health Care Quality Assurance | Data handling; Big Data analytics; Data quality; Health care analytics; Health information technology; Health care claims | Health care data quality | How to assure the data quality while collecting big data form multimodal sources | No | 3 |
| Person-centered health care: a critical assessment of current and emerging research approaches | 2014 | International Journal of Public Health Policy and Health Service Research | complex systems; health services research; individualized care; multimorbidity; paradigm, person-centred health care; systems medicine; transdisciplinary research | Personalized Health care | Focused on ensuring person centered health care by introducing transdisciplinary and complex systems approach that can provide informed decision making framework | Yes | 5 |
| Developing Public Policy To Advance The Use Of Big Data In Health Care | 2014 | Health Affairs | N/A | Policy development using Big data | Finding the aspects of big data that are most relevant to Health care. Exploring the barriers that are challenges to successful implementation of public health policy | Yes | 5 |
| Creating Value In Health Care Through Big Data: Opportunities And Policy Implications | 2014 | Health Affairs | N/A | Data management | Discussed the necessity of proper management and confidentiality of healthcare data along with the benefit of big data analytics | Yes | 5 |
| Big Data In Health Care: Using Analytics To Identify And Manage High Risk And High Cost Patients | 2014 | Health Affairs | N/A | Analytics for high risk patients and Overall Health care | Identifying high risk patients to ensure better care, and also explored the analytics procedure, algorithms and challenges to implement analytics. | Yes | 5 |
| The Legal And Ethical Concerns That Arise From Using Complex Predictive Analytics In Health Care | 2014 | Health Affairs | N/A | Healthcare policy making | Addressed the challenges related to the policy, ethical and legal issues while performing predictive analytics on health care big data | Yes | 5 |
| Transforming Health Care Service Delivery and Provider Selection | 2011 | Journal of digital imaging | Patient empowerment; Quality performance; Data mining | Health Care Service Delivery | Quantifying performance in the delivery of medical services | Yes | 5 |
| Healthy Predictions? Questions for Data Analytics in Health Care | 2016 | American Business Law Journal | N/A | Patient privacy and equality of care provided | Ensuring patient privacy and eliminating discrimination in the health care provided to patient. | No | 4 |
| Sharing State Mental Health Data for Research: Building Toward Ongoing Learning in Mental Health Care Systems | 2015 | Administration and Policy in Mental Health and Mental Health Services Research | Mental health; Health services research;  State data; Data pooling | Mental Health | Mainly addressed the activities executed by National Institute of Mental Health (NIMH) to promote optimal collection, aggregation and use of big data to support mental health service delivery, organization and financing. | Yes | 5 |
| Building a Rapid Learning Health Care System for Oncology: The Regulatory Framework of CancerLinQ | 2014 | Journal of clinical oncology | N/A | Cancer Treatment | Developed a learning Intelligence based network framework to deliver quality care to cancer patients by leveraging the advantages of big data originated from the enormous amount of patients' electronic health records. While doing so, also aimed to maintain the legislative authority of the medicare service to ensure the confidentiality of the patients. | Yes | 5 |
| Big Data, Big Knowledge: Big Data for Personalized Healthcare | 2015 | IEEE journal of biomedical and health informatics | Big data; healthcare; virtual physiological human | Big data analytics framework | Described ﬁve major problems that need to be tackled in order to have an effective integration of big data analytics and VPH modeling in healthcare | Yes | 5 |
| Making sense of big data in health research: Towards an EU action plan | 2016 | Genome Medicine | N/A | Barrier to big data in healthcare | Address barriers for exploitation of health data in Europe | Yes | 5 |
| Big data for health | 2015 | IEEE journal of biomedical and health informatics | Big data; bioinformatics; health informatics; medical  imaging; medical informatics; precision medicine; sensor  Informatics; social health | Recent development and challenges | Discuss recent development in big data health care analytics and challenges faced by healthcare community. | Yes | 5 |
| Translation in Data Mining to Advance Personalized Medicine for Health Equity | 2015 | Intelligent information management | Data Mining; Electronic Medical Records; Translation; Personalized Medicine; Biomedical Informatics; Heath Equity; Healthcare Workforce | Personalized Medicine | This paper looks at the concept of personalized medicine; offering perspectives in four important topic: the availability of "big data" and the ole of biomedical informatics in personalized medicine; the need for interdisciplinary teams in the development and evaluation of personalized therapeutic approaches; the impact of electronic medical record systems and clinical data warehouses on the field of personalized medicine; and an overview of the ethical concerns related to personalized medicine and health equity. | No | 5 |
| SPOC: A secure and privacy preserving opportunistic computing framework for mobile health care emergency | 2013 | IEEE transactions on parallel and distributed systems | Mobile-Healthcare emergency; opportunistic computing; user-centric privacy access control; PPSPC | Mobile Health Care | Ensuring Privacy and security while collecting Personal Health care Information (PHI) | No | 5 |
| Prescription Data Mining and the Protection of Patients' Interests | 2010 | J.L. Med. & Ethics | N/A | Privacy | What strategies are appropriate for data mining from physicians' prescriptions while maintaining the patients privacy within the already established regulations | Yes | 5 |
| Exploring the Potential of Predictive Analytics and Big Data in Emergency Care | 2015 | Annals of Emergency Medicine | N/A | Emergency care | Analyze the opportunity and obstacles of applying predictive analytic and big data to emergency care | Yes | 5 |
| The Person-Event Data Environment: leveraging big data for studies of psychological strengths in soldiers | 2013 | Frontier | big data; psychological strengths; cost analysis; healthcare utilization; personnel data | Psychological strengths of soldiers | Provides an overview of uses of PDE | Yes | 4 |
