# Supplementary material for: A Systematic Review on Healthcare Analytics: Application and Theoretical Perspective of Data Mining
Source: Healthcare (Basel). 2018 May 23;6(2):54. doi: 10.3390/healthcare6020054 (PMC6023432; doi:10.3390/healthcare6020054)
Supplement: Supplementary file 1 [file healthcare-06-00054-s001.zip › healthcare-303796-supplementary/S4_Classification of reviewed papers.docx]

S4. Classification of reviewed papers by analytics type, application area, data type, and data mining techniques.

**Table S4.** Classification of reviewed papers

| Analytics type | Application area | Data type | Data mining technique | References |
| --- | --- | --- | --- | --- |
| Descriptive | Clinical decision making | HG | Classification | [40, 49, 61, 72] |
|  |  | HG | Clustering | [45, 48, 58, 67] |
|  |  | HG | Sequential pattern function | [47] |
|  |  | HG | Association | [55, 64] |
|  |  | SD | Clustering | [45] |
|  | Healthcare administration | HG | Classification | [93, 94, 100, 101, 107, 109, 116] |
|  |  | BT | Classification | [97] |
|  |  | HG | Clustering | [102, 111] |
|  |  | HG | Association | [93, 99, 101] |
|  |  | WS | Clustering | [112, 113] |
|  |  | WS | Association | [115, 117] |
|  |  | WS | Classification | [114] |
|  |  | HG | Data warehousing | [93] |
|  | Privacy and fraud detection | WS | Association | [120] |
|  |  | BT | Classification | [120] |
|  |  | HG | Classification | [122] |
|  | Mental health | HG | Classification, Association | [126] |
|  |  | HG | Clustering | [128] |
|  | Pharmacovigilance | WS | Clustering | [140] |
|  |  | HG | Association | [138, 139, 141-144, 146] |
|  |  | BT | Association | [145] |
|  | Public health | HG | Classification | [130, 134, 136] |
|  |  | HG | Clustering | [134, 135] |
|  |  | HG | Data warehousing | [130] |
|  |  | HG | Association | [134] |
|  |  | WS | Association | [133] |
| Predictive | Clinical decision making | HG | Classification | [38, 41-44, 46, 50-54, 56, 57, 59, 60, 62, 63, 65, 69-73, 75, 76] |
|  |  | HG | Association | [57] |
|  |  | HG | Sequential pattern mining | [74] |
|  |  | WS | Classification | [65] |
|  |  | HG | Clustering | [53, 66] |
|  |  | SD | Classification | [46, 71] |
|  |  | BM | Classification | [68] |
|  |  | BT | Classification | [44] |
|  | Healthcare administration | HG | Classification | [98, 100, 103, 105, 107, 110] |
|  |  | HG | Clustering | [95, 110] |
|  |  | HG | Regression | [108] |
|  |  | SD | Classification | [105] |
|  |  | BT | Classification | [96] |
|  |  | BT | Clustering | [96] |
|  |  | WS | Regression | [108, 114] |
|  |  | WS | Classification | [104] |
|  | Mental health | HG | Classification | [128] |
|  | Public health | HG, SD | Classification | [131] |
| Prescriptive | Healthcare administration | HG | Association | [106] |
|  |  | HG | Data warehousing | [89-92] |
|  | Privacy and fraud detection | HG | Data warehousing | [119] |
|  | Mental health | SD | Anomaly detection | [127] |
|  |  | HG | Clustering, Classification | [125] |
|  | Public health | HG | Classification, Clustering | [132] |

Human generated data = HG, Web/ social media data = WS, Sensor data = SD, Biometric data = BM, Big transection data = BT
